# Star nanoparticles delivering HIV-1 peptide minimal immunogens elicit near-native envelope antibody responses in nonhuman primates

**DOI:** 10.1371/journal.pbio.3000328

**Published:** 2019-06-17

**Authors:** Joseph R. Francica, Richard Laga, Geoffrey M. Lynn, Gabriela Mužíková, Ladislav Androvič, Baptiste Aussedat, William E. Walkowicz, Kartika Padhan, Ramiro Andrei Ramirez-Valdez, Robert Parks, Stephen D. Schmidt, Barbara J. Flynn, Yaroslav Tsybovsky, Guillaume B. E. Stewart-Jones, Kevin O. Saunders, Faezzah Baharom, Constantinos Petrovas, Barton F. Haynes, Robert A. Seder

**Affiliations:** 1 Vaccine Research Center, National Institute of Allergy and Infectious Diseases, National Institutes of Health, Bethesda, Maryland, United States of America; 2 Institute of Macromolecular Chemistry, Czech Academy of Sciences, Prague, Czech Republic; 3 Avidea Technologies, Inc., Baltimore, Maryland, United States of America; 4 Department of Chemical Biology, Memorial Sloan Kettering Cancer Center, New York, New York, United States of America; 5 Duke Human Vaccine Institute, Duke University School of Medicine, Durham, North Carolina, United States of America; 6 Electron Microscopy Laboratory, Cancer Research Technology Program, Leidos Biomedical Research Inc., Frederick National Laboratory for Cancer Research, Frederick, Maryland, United States of America; University of Michigan, UNITED STATES

## Abstract

Peptide immunogens provide an approach to focus antibody responses to specific neutralizing sites on the HIV envelope protein (Env) trimer or on other pathogens. However, the physical characteristics of peptide immunogens can limit their pharmacokinetic and immunological properties. Here, we have designed synthetic “star” nanoparticles based on biocompatible *N*-[(2-hydroxypropyl)methacrylamide] (HPMA)-based polymer arms extending from a poly(amidoamine) (PAMAM) dendrimer core. In mice, these star nanoparticles trafficked to lymph nodes (LNs) by 4 hours following vaccination, where they were taken up by subcapsular macrophages and then resident dendritic cells (DCs). Immunogenicity optimization studies revealed a correlation of immunogen density with antibody titers. Furthermore, the co-delivery of Env variable loop 3 (V3) and T-helper peptides induced titers that were 2 logs higher than if the peptides were given in separate nanoparticles. Finally, we performed a nonhuman primate (NHP) study using a V3 glycopeptide minimal immunogen that was structurally optimized to be recognized by Env V3/glycan broadly neutralizing antibodies (bnAbs). When administered with a potent Toll-like receptor (TLR) 7/8 agonist adjuvant, these nanoparticles elicited high antibody binding titers to the V3 site. Similar to human V3/glycan bnAbs, certain monoclonal antibodies (mAbs) elicited by this vaccine were glycan dependent or targeted the GDIR peptide motif. To improve affinity to native Env trimer affinity, nonhuman primates (NHPs) were boosted with various SOSIP Env proteins; however, significant neutralization was not observed. Taken together, this study provides a new vaccine platform for administration of glycopeptide immunogens for focusing immune responses to specific bnAb epitopes.

## Abstract 2

Synthetic polymer-based nanoparticles effectively deliver HIV Env glycopeptide immunogens to lymph nodes and stimulate B cell lineages with characteristics resembling broadly neutralizing antibodies, in nonhuman primates.

## Introduction

A major goal of HIV vaccine development is to elicit broadly neutralizing antibodies (bnAbs) [1] capable of preventing infection from the wide genetic diversity of circulating HIV-1 strains [2–4]. bnAbs can arise during chronic HIV infection in humans [5–7], and such antibodies can prevent infection upon passive transfer to NHPs [8–10]. These findings provide compelling evidence that a preventive vaccine for HIV may be attainable if immunogens can be designed to elicit bnAbs.

One critical step toward designing such immunogens was the discovery of a method to stabilize HIV envelope protein (Env) trimers in their native conformation (termed SOSIP, for critical stabilizing mutations [11]). bnAbs preferentially bind to SOSIP trimers, compared with strain-specific monoclonal antibodies (mAbs) [12], and vaccine studies have shown they can elicit autologous neutralizing responses in NHPs, although reproducible induction of breadth has not yet been observed [13–17]. Several factors may play a role in limiting the induction of neutralization breadth. Poor binding of SOSIP trimer immunogens to bnAb precursors, called unmutated common ancestors (UCAs), results in limited priming of the necessary B cell lineages [18], although this may improve with next-generation SOSIP immunogens [19]. Another limitation may be that full-length Env trimers present an array of immunodominant epitopes, including non-neutralizing or strain-specific neutralizing epitopes, which could limit bnAb responses [20].

Several current vaccine approaches are attempting to focus the immune response to specific Env bnAb epitopes, called sites of vulnerability [21]. One such approach uses an immunogen engineered from the Env outer domain, termed eOD [22]. This presents the bnAb epitope of the cluster of differentiation 4 (CD4) binding site (CD4bs) and was designed to bind with high avidity to bnAb UCAs targeting this region [23]. This single-domain immunogen was multimerized on recombinant nanoparticles and was able to prime appropriate B cells in transgenic mice [24, 25]. The eOD is a promising approach because it presents fewer off-target epitopes than full Env trimers and also because it has been designed to engage bnAb UCAs. Nonetheless, the elicitation of CD4bs bnAbs remains a challenging goal, because CD4bs bnAbs are observed to have very high levels of somatic hypermutation (SHM) of around 30% [26], which may be required for neutralization breadth [27].

A second approach for focusing responses to Env sites of vulnerability is to use peptide minimal immunogens. Such immunogens mimic bnAb epitopes with minimal surrounding protein structure to minimize off-target responses. Fully synthetic glycopeptide immunogens have been created for the HIV Env variable loop 1 and 2 (V1V2)/glycan site [28] and the Env variable loop 3 (V3)/glycan site [29, 30]. The fusion peptide site may also be amenable to such immunogen design because of its small size [31, 32]. These glycopeptide minimal immunogens have also been shown to bind to bnAb UCAs, suggesting they may be useful for priming bnAb lineages [33, 34].

While peptide minimal immunogens may be useful for focusing the immune response, their small size makes them poorly immunogenic because of unfavorable pharmacokinetic properties and their inability to cross-link B cell receptors (BCRs). Therefore, in this study we have created a vaccine delivery platform for synthetic minimal immunogens that would improve their immunogenicity and can be linked directly to potent Toll-like receptor (TLR) 7/8 agonist adjuvants [35]. We focused here on minimal immunogens that target the V3/glycan site of vulnerability because V3/glycan bnAbs only require a moderate level of SHM to achieve neutralization breadth [34, 36], which is a major limitation to inducing bnAbs to other sites of vulnerability. Accordingly, V3/glycan bnAbs have been found to neutralize approximately 60% of tier 2 viral strains with only 15%–20% SHM [37].

We have previously shown that synthetic polymer-based vaccine platforms can be used to co-deliver small molecule adjuvants with immunogens, leading to protective T-cell and antibody responses [38, 39]. Here, a new approach using a synthetic nanoparticle platform based on a poly(amidoamine) (PAMAM) dendrimer core grafted with poly[N-(2-hydroxypropyl) methacrylamide] (PHPMA) extensions was designed to display peptide minimal immunogens using a covalent attachment. We characterized the nanoparticles’ pharmacokinetics and antigen-presenting cell (APC) uptake, and demonstrated how altering their physical properties allows for the optimization of vaccine immunogenicity. We then created nanoparticles displaying an Env variable loop 3 oligomannose-9 glycopeptide (Man_9_V3) minimal immunogen targeting the V3/glycan site of vulnerability [29, 34]. Immunizations in NHP were performed to focus the response to the V3/glycan site, followed by two SOSIP Env boosting strategies. While these particular boosting regimens were not sufficient for neutralization, this nanoparticle platform provides a method of focusing the immune response to a particular Env site of vulnerability.

## Results

### Design of a vaccine delivery platform to improve the immunogenicity of a minimal peptide immunogen

Peptide minimal immunogens have been designed to mimic multiple epitopes on the HIV Env trimer. However, because of their small size, they may be poorly immunogenic. Immunization of mice with a prototype peptide immunogen derived from the crown of the HIV V3 loop [40] with a universal CD4 T-cell helper peptide (pan DR epitope [PADRE]) resulted in low or undetectable antibody titers, even when admixed with potent commercially available adjuvants: Sigma Adjuvant System (Ribi), AddaVax (an emulsion similar to MF59), polyinosinic-polycytidylic acid (polyIC:LC), or alum (S1 Fig). Therefore, we undertook the design of a vaccine delivery platform that would improve the immunogenicity of peptide minimal immunogens when formulated with these common adjuvants.

**Fig 1:**
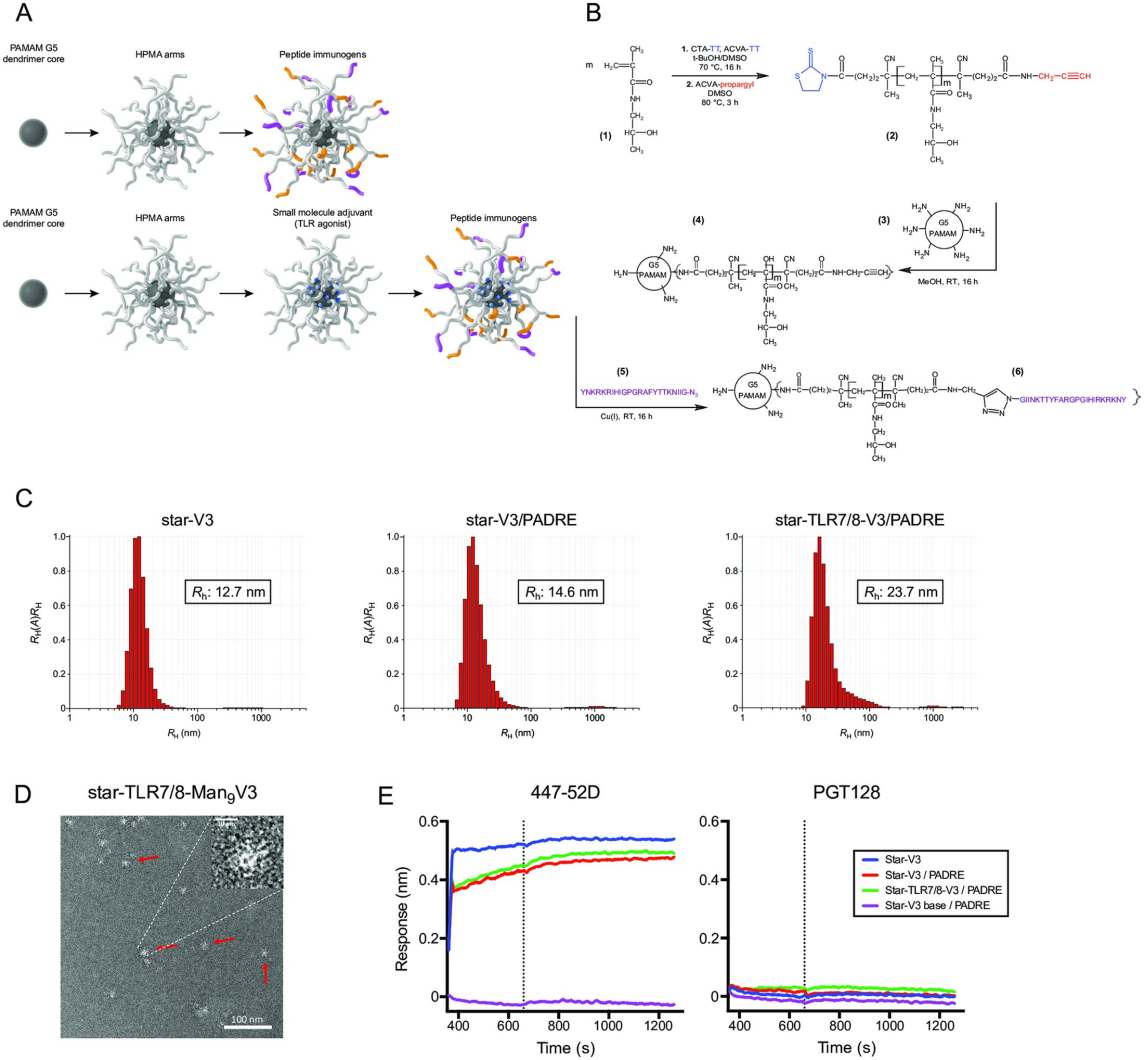
Star nanoparticle synthesis and characterization. (*A*) Cartoon depiction of star nanoparticles, composed of a dendrimer core and HPMA-based arms, to which peptide immunogens (yellow and purple) are attached. Small molecule adjuvants may also be attached, shown as blue polygons in the lower row scheme. (*B*) Star nanoparticles are synthesized by polymerization of HPMA monomers (**1**) into 10-kDa chains (**2**), then conjugating these to G5 PAMAM dendrimers by acylation (**3**) to yield unloaded nanoparticles (**4**); finally, peptide immunogens (**5**) are conjugated using to the HPMA grafts by Cu^I^ catalyzed cycloaddition to yield the loaded vaccine nanoparticle (**6**). (*C*) Dynamic light scattering analysis of star nanoparticles displaying monodispersity, with hydrodynamic radius (*R*_h_) shown. (*D*) Star nanoparticles bearing a TLR7/8 agonist and glycosylated V3 peptides (Man_9_V3, described in Fig 6) as visualized by electron microscopy, magnification 100,000×; red arrows indicate individual nanoparticles. (*E*) The antigenicity of star nanoparticles bearing HIV V3 and PADRE peptides and a TLR7/8 agonist was analyzed by biolayer interferometry. Antibodies 447-52D (specific for V3 loop apex) and PGT128 (specific for glycan and V3 loop base) were immobilized on sensors; binding of star nanoparticles in solution was then measured. Nanoparticles displaying a V3 epitope comprising the unglycosylated loop base (purple trace) serve as a negative control. Data associated with this figure can be found in S1 Data. ACVA, 4,4′-azobis(4-cyanovaleric acid); Cu^I^, copper(I); G5, fifth-generation; HPMA, *N*-[(2-hydroxypropyl)methacrylamide]; Man_9_V3, Env variable loop 3 oligomannose-9 glycopeptide; PADRE, pan DR epitope; PAMAM, poly(amidoamine); TLR7/8, Toll-like receptor 7/8; V3, Env variable loop 3.

To improve the immunogenicity of peptide minimal immunogens, we designed a completely synthetic nanoparticle vaccine based on PAMAM dendrimer cores, with PHPMA arms extending from the core, to which immunogens and TLR adjuvants could be covalently attached (Fig 1A and 1B; S2 Fig). Based on their appearance, these nanoparticles are referred to as “star” nanoparticles. The star nanoparticle platform is modular, allowing physical parameters such as particle size, antigen density, and adjuvant attachment to be changed by varying the molar ratios and molecular weights of the components. We found that the combination of a fifth-generation (G5) PAMAM dendrimer with approximately 10 kDa PHPMA arms at a molar ratio of 2:1 (PAMAM primary amino groups to PHPMA thiazolidine-2-thione [TT] groups) produced stable approximately 20-nm nanoparticles with up to 33 arms for immunogen display. Unreacted amino groups were still present on the dendrimer surface that could be used for adjuvant attachment (S3 Fig). As expected, lower-generation dendrimers resulted in nanoparticles with fewer PHPMA arms. Interestingly, the use of larger molecular weight PHPMA arms reduced the number of peptide immunogens that could be conjugated, presumably because of steric hindrance. The PHPMA polymer arms serve to eliminate dendrimer positive charge, resulting in the zeta potential of the star nanoparticles of zero.

All parameters for the star nanoparticle conjugates used in this study are described in S1 Table. Using a prototypic V3 peptide immunogen, the hydrodynamic radius of star nanoparticles was found to be 13 nm by dynamic light scattering (approximately 30 V3 peptides/nanoparticle). Similar measurements were obtained when a mixture of V3 and PADRE T-helper peptides were attached (15 nm; approximately 15 V3 peptides and 15 PADRE peptides/nanoparticle), although the radius increased slightly when a small molecule TLR7/8 agonist adjuvant was attached to the core (24 nm), suggesting a conformational difference in the flexible *N*-[(2-hydroxypropyl)methacrylamide] (HPMA) arms (Fig 1C). Electron microscopy confirmed the overall shape of star nanoparticles to be semispherical (Fig 1D). Finally, biolayer interferometry (BLI) indicated that star nanoparticles bearing V3 peptides could be readily recognized by the V3 loop crown antibody, 447-52D [41]; nanoparticles bearing a peptide from the V3 loop base did not bind, as expected. These data show that V3 peptides displayed on star nanoparticles retained their antigenicity and are accessible at the particles’ surface.

### Pharmacokinetics of star nanoparticles

**Fig 2:**
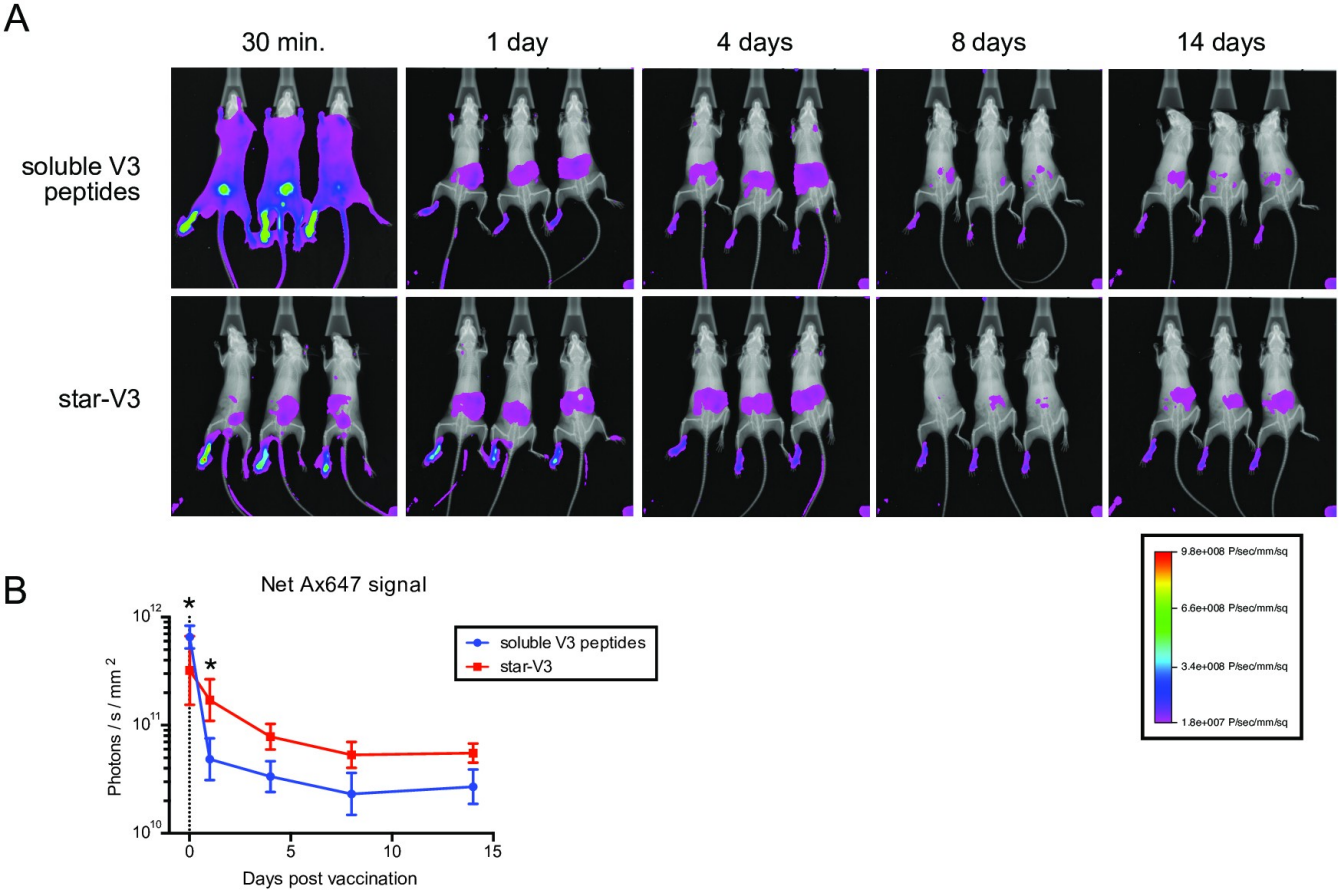
Star nanoparticles restrict the biodistribution and prolong the localization of minimal peptide immunogens. Mice were immunized subcutaneously in the left footpad with star nanoparticles bearing Ax647-labeled V3 peptides; control mice were immunized with soluble Ax647-labeled V3 peptides. (*A*) Mice were imaged at the indicated time points following vaccination. Composite overlays of X-ray and fluorescent images are shown. (*B*) Vaccine retention at the site of injection was measured by quantifying fluorescence in the left footpad at the time points shown above. Data points indicate group geometric means and 95% confidence intervals; vertical line indicates immunization; asterisk (*), statistical difference by ANOVA, comparing between groups at each time point. Data associated with this figure can be found in S1 Data. Ax647, Alexa Fluor 647; V3, Env variable loop 3.

To evaluate the pharmacokinetic effects of arraying peptide minimal immunogens on the surface of star nanoparticles, these were visualized following immunization in mice (Fig 2A). As a control, unconjugated, soluble V3 peptides were found to rapidly disseminate throughout the body by 30 minutes postinjection. For the remainder of the 2-week observation period, the soluble peptides had a biodistribution localized mostly to the liver and spleen and, to a lesser extent, the site of injection. Strikingly, when arrayed on star nanoparticles, the V3 peptides did not show a disseminated biodistribution at any time point but could only be visualized at the injection site and at the liver and spleen region. Because soluble V3 peptides could rapidly diffuse from the site of injection, we quantified the signal in the footpad region over time. Indeed, there was consistently more immunogen remaining at the site of injection over time in the star nanoparticle group compared with the free peptides (Fig 2B). These data demonstrate how nanoparticles can be used to control the pharmacokinetics of peptide immunogens and depict a prolonged localization to the site of injection, which may improve vaccine uptake and immunogenicity.

### Uptake of star nanoparticles in draining lymph nodes following immunization

**Fig 3:**
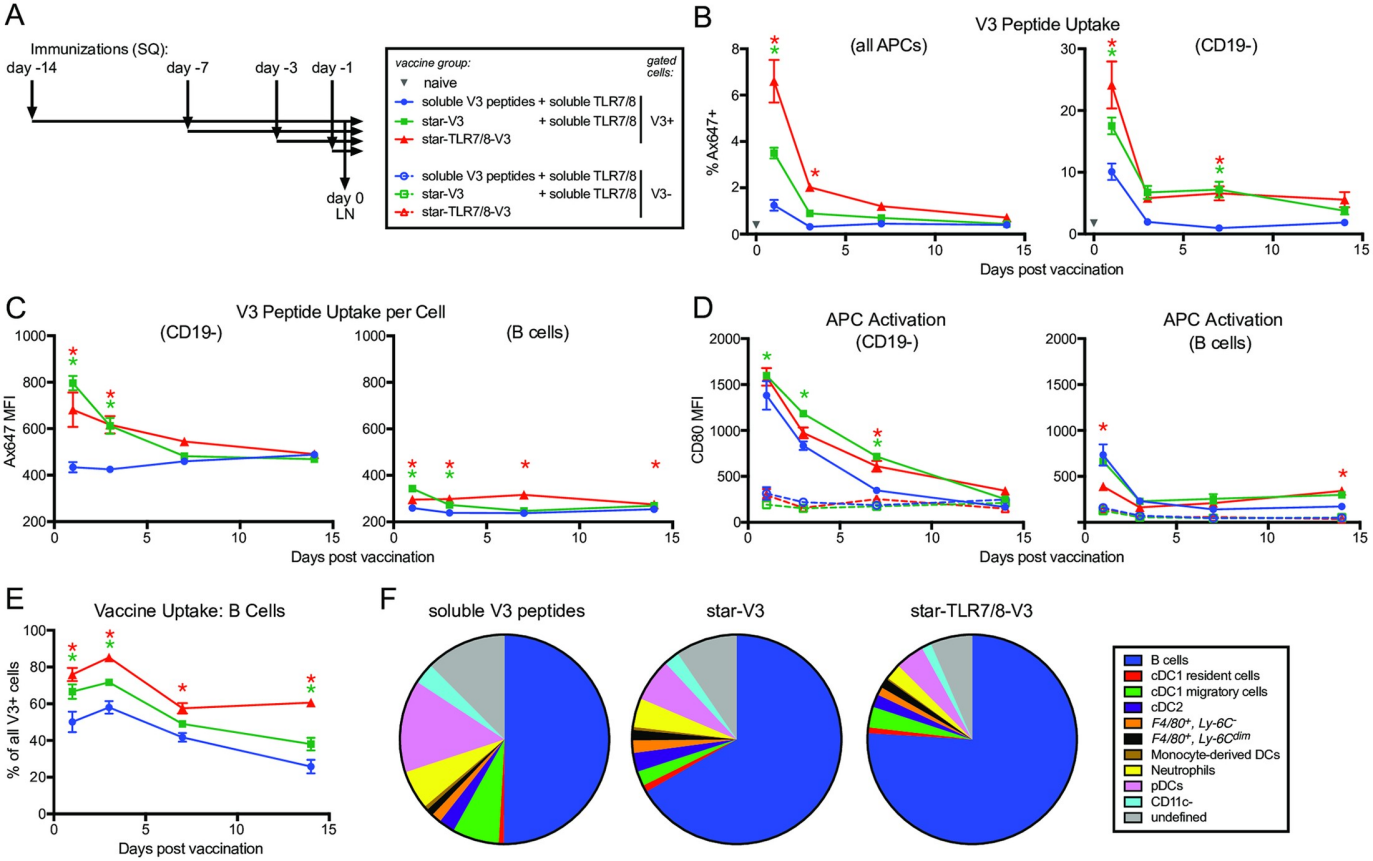
APC uptake of star nanoparticles in the draining LN. Mice were immunized subcutaneously with star nanoparticles bearing Ax647-labeled V3 peptides formulated with a TLR7/8 adjuvant via direct conjugation (red symbols), or admixed with soluble TLR7/8 (green symbols); control mice were immunized with soluble V3 peptides and a soluble TLR7/8 agonist (blue symbols). T-cell help was provided via unlabeled nanoparticles bearing PADRE peptides to all groups. (*A*) Immunization scheme: vaccines were administered 14, 7, 3, or 1 day(s) before day 0; on day 0, popliteal LNs were harvested and analyzed by flow cytometry. (*B*) V3 peptide uptake was measured by gating on Ax647+ cells. Left graph shows positive cells from all APCs; right graph shows non–B cell APCs only. (*C*) V3 peptide uptake per cell was measured by Ax647 MFI in the V3+ gate. Left graph shows non–B cell APCs only; right graph shows B cells only. (*D*) APC activation, as measured by CD80 expression. Left graph shows non–B cell APCs only; right graph shows B cells only. (*E*) Vaccine uptake by B cells, graphed as a percentage of the total number of Ax647+ cells. Asterisk (*), statistical difference by ANOVA, compared with the soluble V3 peptides group for star-V3 group (green) or star-TLR7/8-V3 group (red). (*F*) Vaccine uptake by APC phenotype, 24 hours after vaccination. Data associated with this figure can be found in S1 Data. APC, antigen-presenting cell; Ax647, Alexa Fluor 647; CD, cluster of differentiation; cDC, conventional dendritic cell; LN, lymph node; MFI, median fluorescence intensity; PADRE, pan DR epitope; pDC, plasmacytoid dendritic cell; SC, subcutaneous; TLR7/8, Toll-like receptor 7/8; V3, Env variable loop 3.

To determine the uptake of star nanoparticles by immune cells in vivo, we used flow cytometry to analyze the innate immune response in the draining lymph node (LN) following vaccination (Fig 3A). Consistent with the data in Fig 2, uptake of star nanoparticles was highest at 24 hours postvaccination and then decreased over time. Star nanoparticles were preferentially taken up by APCs (approximately 4%) compared with soluble V3 peptides (approximately 1%) (Fig 3B). However, uptake was further improved with star nanoparticles bearing a TLR7/8 agonist (approximately 6%). We next measured the amount of antigen that was taken up on a per cell basis and found that vaccination with star nanoparticles resulted in approximately twice the amount of uptake per cell at 24 hours, compared with soluble V3 peptides (Fig 3C). Notably, this effect was only seen in CD19− APCs; B cells took up much less of the vaccine per cell, with no difference between soluble peptides or star nanoparticles. A comparison of the co-stimulatory marker CD80 revealed that vaccine+ APCs were significantly more activated compared with vaccine negative cells (Fig 3D). Activation in vaccine+ CD19− APCs was highest at 24 hours and remained elevated until after 7 days postvaccination, while vaccine+ B cells were only activated at 24 hours. Compared with soluble peptides, star nanoparticles induced a consistent and statistically higher activation level over time. Finally, we analyzed the phenotypic makeup of the vaccine+ APCs. The most frequent cell type was B cells, which comprised about 70%–80% of APCs taking up star nanoparticles, compared with only 50% of those taking up soluble peptides (Fig 3E). This low-level nonspecific B cell uptake of star nanoparticles bearing a TLR7/8 agonist accounts for the improved overall APC uptake shown in Fig 3B. The remaining vaccine+ cell types were distributed among various dendritic cell (DC)–related, monocyte-related, and other cell subsets (Fig 3F). Interestingly, a significant proportion of plasmacytoid DCs were positive for soluble V3 peptides.

**Fig 4:**
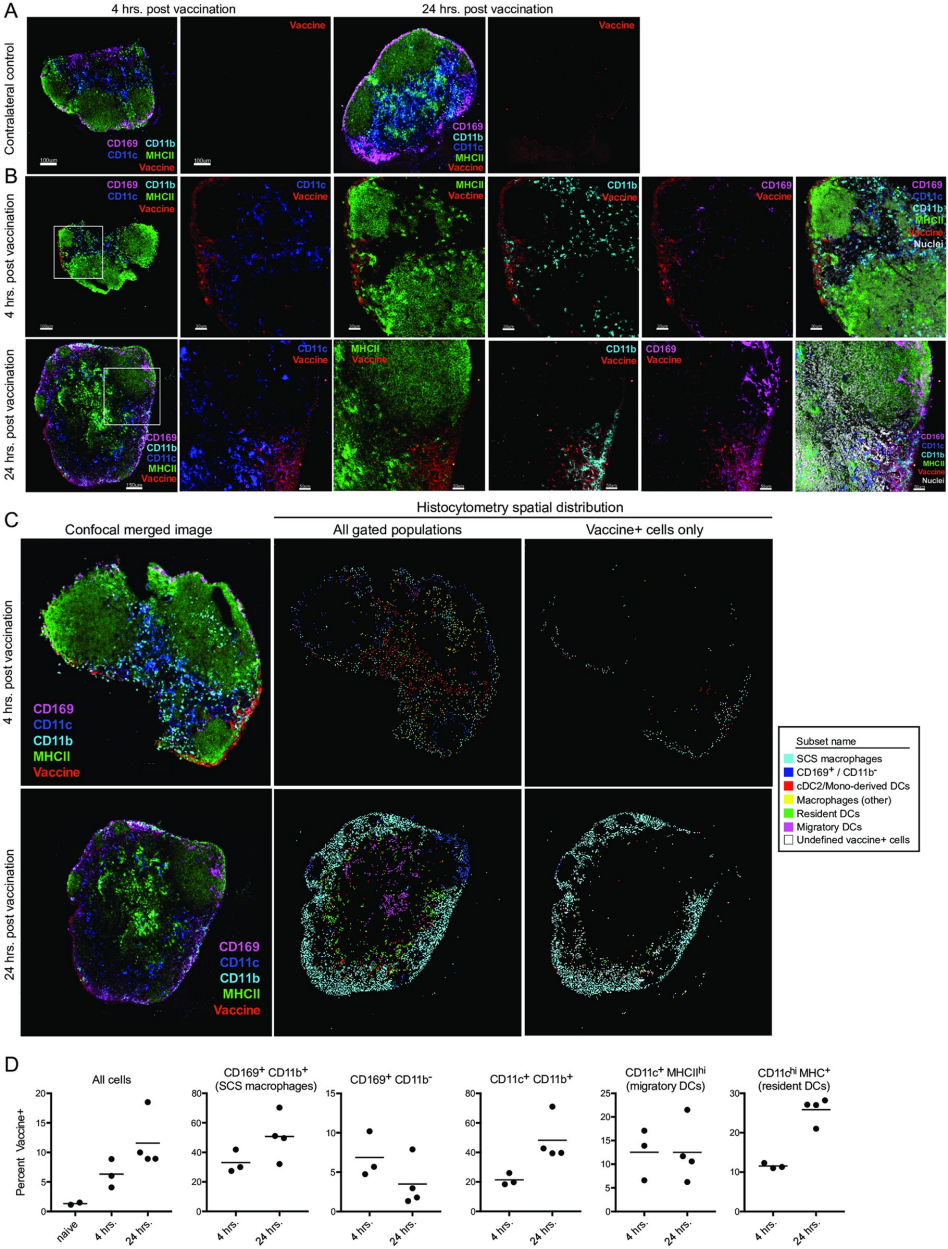
Star nanoparticle spatial distribution on APCs in draining LNs. Mice were vaccinated in the footpad, with star nanoparticles bearing Ax647-labeled V3 peptides and unlabeled nanoparticles bearing PADRE peptides and a soluble TLR7/8 agonist. (*A*) Confocal microscopy of contralateral control LNs at 4 and 24 hours or (*B*) vaccinated draining ipsilateral LNs at 4 hours (top row) or 24 hours (bottom row). Colocalization of the vaccine with various APC markers is shown. (*C*,*D*) Histocytometry analysis of vaccine+ APC populations. (*B*) Confocal images from (*B*, left panels) were gated into the indicated populations based on marker co-expression, then replotted by their x- and y- coordinates. Center panels depict all gated subsets; right panels show only vaccine+ cells. (*D*) Vaccine+ events are graphed as a percentage of each gated population, from draining LNs of multiple mice. Data associated with this figure can be found in S1 Data. APC, antigen-presenting cell; Ax647, Alexa Fluor 647; CD, cluster of differentiation; cDC, conventional dendritic cell; DC, dendritic cell; LN, lymph node; MHCII, major histocompatibility complex class II; PADRE, pan DR epitope; SCS, subcapsular sinus; TLR7/8, Toll-like receptor 7/8; V3, Env variable loop 3.

To determine the spatial distribution of star nanoparticles after uptake by APCs, we visualized vaccine draining LNs by confocal microscopy. Star nanoparticles could be detected 4 hours postvaccination in the subcapsular sinus (SCS) and—to a lesser extent—the cortex, which colocalized with various APC markers (Fig 4A and 4B). Histocytometry was then used to spatially analyze vaccine+ APC subsets (S5 Fig). At 4 and 24 hours postvaccination, vaccine+ cells were mostly CD169^+^ CD11b^+^ SCS macrophages (Fig 4C). As expected, these cells ware localized to the capsule and are known to take up viral pathogens and present them to B cells [42]. Interestingly, vaccination induced a disruption and relocalization of these SCS macrophages from their peripheral distribution (S6A Fig). A similar disruption has been reported in response to inflammation [43], and these cells are known to respond to TLR4 agonists [44]. However, this phenomenon has yet to be reported following immunization with a TLR7/8 agonist, which we found to be necessary to trigger SCS macrophage disruption (S6B Fig). Vaccine colocalization was less prominently observed with CD11c^+^ major histocompatibility complex class II (MHCII)^hi^ migratory DCs, but vaccine+ CD11c^hi^ MHCII^+^ resident DCs increased from 4 to 24 hours postvaccination, suggesting the nanoparticles were being transferred to other APCs (Fig 4D). Taken together, these data demonstrate how readily star nanoparticles traffic to the LN, where they are taken up by various immune cell types.

### Optimization of star nanoparticle immunogenicity

**Fig 5:**
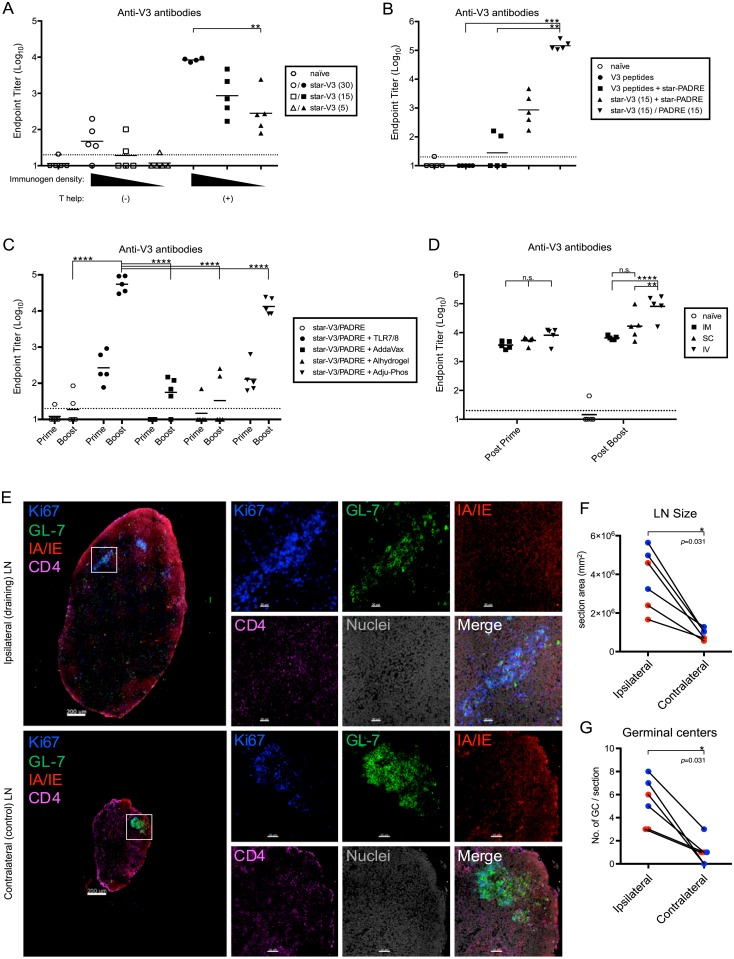
Optimization of nanoparticle immunogenicity. (*A*) Mice were immunized subcutaneously with star nanoparticles bearing 5, 15, or 30 V3 peptides per particle, with or without star nanoparticles bearing PADRE peptides for T help. The V3 peptide dose (5 μg) was constant across all groups; all vaccines were adjuvanted by admixing with a soluble TLR7/8 agonist. (*B*) Mice were immunized with soluble V3 peptides, with or without star nanoparticles bearing PADRE peptides, with star nanoparticles bearing V3 or PADRE peptides on separate particles, or with star nanoparticles bearing both V3 and PADRE peptides on the same particle; the density of V3 peptides (15/nanoparticle) was constant across all groups; the V3 peptide dose (5 μg) was constant across all nanoparticle groups; all vaccines were adjuvanted by admixing with soluble a TLR7/8 agonist. (*C*) Comparison of different adjuvants for use with star nanoparticles. Nanoparticles bearing V3 and PADRE peptides were left unadjuvanted, or were admixed with a TLR7/8 agonist, the emulsion adjuvant AddaVax, or the alum formulations Alhydrogel or Adju-Phos. (*D*) Comparison of different vaccination routes. Star nanoparticles bearing TLR7/8 agonists, V3, and PADRE peptides were administered intramuscularly (IM), subcutaneously (SC), or intravenously (IV). In all studies, serum antibody responses were measured by ELISA after two homologous immunizations, except where indicated; bars indicate group geometric means; horizontal lines indicate ELISA assay limit of detection. *p < 0.05; **p < 0.01; ***p < 0.001; ****p < 0.0001; n.s., not significant by the Kruskal-Wallis test. (*E-G*) Germinal center formation in popliteal LNs 10 days after footpad immunization, with star nanoparticles bearing Ax647-labeled V3 peptides and unlabeled nanoparticles bearing PADRE peptides and a soluble TLR7/8 agonist. (*E*) Germinal centers are visualized by confocal microscopy with Ki67 and GL-7 staining in the vaccinated draining (ipsilateral) or unvaccinated (contralateral) LNs; scale bars indicate 200 μm and 30 μm, respectively. (*F*) General inflammation was quantified by measuring the size of ipsilateral and contralateral LNs. (*G*) Germinal centers were enumerated in the ipsilateral and contralateral LNs. Red and blue symbols indicate samples from two independent experiments; *p*-values are derived from the Wilcoxon matched-pairs signed-rank test. Data associated with this figure can be found in S1 Data. Ax647, Alexa Fluor 647; CD, cluster of differentiation; GC, germinal center; IM, intramuscularly; IV, intravenously; LN, lymph node; n.s., not significant; PADRE, pan DR epitope; SC, subcutaneously; TLR7/8, Toll-like receptor 7/8; V3, Env variable loop 3.

We next optimized immunogenicity by varying the density of peptide immunogens and the presence of T-helper peptides on the star nanoparticles. Keeping a constant immunogen dose across all groups, mice were immunized with star nanoparticles bearing 5, 15, or 30 V3 peptides per particle (Fig 5A). Binding antibody titers were directly correlated with antigen density. The importance of multimerization around the dendrimer core was further supported by the observation that unattached linear PHPMA arms alone bearing an average of either 4.3 or 11.5 V3 peptides per chain were less efficient in eliciting V3 antibodies compared with star nanoparticles (S7 Fig)

We next attempted to further improve antibody titers by directly targeting T-helper cells with PADRE [45]. While soluble V3 peptides were non-immunogenic and could not be significantly improved by the addition of star nanoparticles containing PADRE peptides, a mixture of star nanoparticles displaying either V3 or PADRE peptides was immunogenic (Fig 5B). Interestingly, star nanoparticles displaying 15 V3 peptides and 15 PADRE peptides on the same nanoparticle elicited V3 titers that were 2 logs higher than the separate nanoparticles. These co-mixed particles were also found to improve PADRE-specific T-cell responses, compared with separate particles (S8A Fig). These data established that a G5 dendrimer with 10-kD HPMA arms displaying a mixture of V3 (or other minimal immunogen) and PADRE peptides at the maximum loading density best induced antibody responses in mice.

We next examined how several different adjuvants that have been used clinically altered the antibody response elicited by star nanoparticles. Consistent with prior studies by us and others [46, 47], the TLR7/8 agonist was found to be the most potent of all adjuvants tested, followed by Adju-Phos (Fig 5C). Of note, AddaVax and Alhydrogel had limited activity. The increase in antibody titers by Adju-Phos compared with Alhydrogel is likely because the V3 and PADRE peptides are positively charged, while Adju-Phos carries a negative charge that may facilitate its association with the star nanoparticles; Alhydrogel carries a positive charge that could repel the nanoparticles.

We also compared the route of administration using star nanoparticles bearing V3 and PADRE peptides, with the TLR7/8 agonist attached to the dendrimer core. Direct conjugation of the TLR7/8 agonist prevents the separation of the adjuvant from the nanoparticles after vaccination due to different pharmacokinetics and allows for synchronous delivery of antigen and innate stimulation [48]. Mice were immunized intramuscularly (IM), subcutaneously (SC), and intravenously (IV) (Fig 5D). While no difference was observed after one immunization, mice immunized by the IV route had approximately 1 log higher titers than the IM and SC groups after a boost. IV administration also resulted in higher splenic PADRE T-cell responses (S8B Fig).

Finally, we visualized the induction of germinal center responses by confocal microscopy. Draining popliteal LNs in mice vaccinated with star nanoparticles and a TLR7/8 agonist were substantially enlarged compared with contralateral, control LNs on the unvaccinated side (Fig 5E and 5F). Germinal centers were identified as GL-7+/Ki67+ clusters, which were consistently more numerous in vaccinated draining LNs, compared with contralateral control LNs (Fig 5G).

### Design of nanoparticles to prime V3-directed neutralizing antibodies

**Fig 6:**
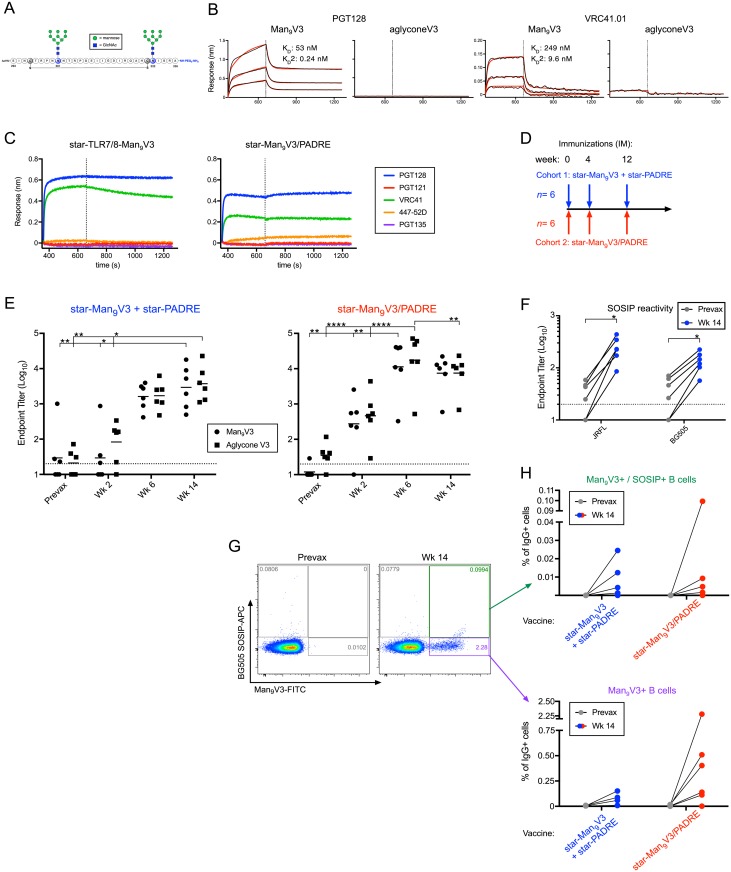
Immunogenicity of star nanoparticles in NHP. (*A*) The synthetic minimal peptide immunogen, Man_9_V3, was designed to mimic the epitopes bound by the bnAbs PGT128 and VRC41. (*B*) Binding affinity was measured by BLI, in which the biotinylated glycosylated (Man_9_) or unglycosylated (aglycone) peptide immunogens were immobilized and then associated with PGT128 (left graph) or VRC41.01 (right graph). Vertical lines indicate the transition from association to dissociation phases. (*C*) The antigenicity of the star nanoparticles was confirmed by BLI, in which various V3-directed mAbs were immobilized and then associated with star nanoparticles bearing Man_9_V3 alone (left graph) or with PADRE (right graph). (*D*) NHPs were homologously immunized in two cohorts at 0, 4, and 12 weeks. The first cohort was given a mixture of star nanoparticles bearing either Man_9_V3 or PADRE peptides. The second cohort was given nanoparticles bearing both Man_9_V3 and PADRE peptides. (*E*) Serum binding titers measured after each immunization to ELISA plates coated with Man_9_V3 or aglycone (non-glycosylated) V3 peptides. Cohort 1, left graph; cohort 2, right graph. Bars indicate group geometric means; horizontal lines indicate ELISA assay limit of detection. *p < 0.05, **p < 0.01, ****p < 0.0001 by the Kruskal-Wallis test. (*F*) Serum binding titers from cohort 1 measured after the third immunization to ELISA plates coated with JRFL SOSIP trimers. *p < 0.05 by the Wilcoxon test. (*G*,*H*) B cell immunophenotyping to identify vaccine-specific responses (Man_9_V3 probe) and cross-reactive SOSIP responses (BG505 SOSIP probe). (*G*) Representative flow cytometry plots showing prevaccination and peak vaccine responses. (*H*) B cell immunophenotyping by cohort for vaccine-specific responses (bottom graph) and cross-reactive SOSIP responses (top graph). Data associated with this figure can be found in S1 Data. APC, antigen-presenting cell; BLI, biolayer interferometry; bnAb, broadly neutralizing antibody; GlcNAc, *N*-acetylglucosamine; IgG+, immunoglobulin G; mAb, monoclonal antibody; Man_9_V3, Env variable loop 3 oligomannose-9 glycopeptide; NHP, nonhuman primate; PADRE, pan DR epitope; TLR7/8, Toll-like receptor 7/8; V3, Env variable loop 3.

Having optimized a nanoparticle vaccine to deliver peptide minimal immunogens, we undertook a series of NHP studies to evaluate whether they could be used to elicit neutralizing antibody responses to HIV. For these studies, a minimal immunogen designed to mimic the V3/glycan bnAb epitope recognized by PGT128, VRC41, and others was used. This synthetic immunogen, Man_9_V3, was derived from the JRFL strain and contains two high mannose glycan moieties at positions 301 and 332 (Fig 6A) that enable it to be recognized by PGT128 and VRC41 with nanomolar affinity (Fig 6B and [29]). The Man_9_V3 glycopeptide immunogen was then arrayed onto star nanoparticles with or without PADRE peptides. These nanoparticles still bound to PGT128 and VRC41 by BLI (Fig 6C). PGT121, which prefers Man_5_ sugars, was not bound, nor was 447-52D, whose immunodominant V3 crown epitope was removed from this construct to prevent off-target effects. These data show that after conjugation to star nanoparticles, the antigenicity of the Man_9_V3 immunogen was preserved.

Rhesus macaques were immunized at 0, 4, and 12 weeks in two cohorts, receiving either Man_9_V3 and PADRE immunogens in separate (cohort 1) or mixed (cohort 2) nanoparticles (Fig 6D) admixed with the TLR7/8 agonist adjuvant. For the first immunization in cohort 1, a low dose of the immunogen and adjuvant was used while monitoring for any adverse events; none were observed. Dosing was then increased for subsequent immunizations (S2 Table). High plasma antibody titers were elicited after two immunizations, with cohort 2 trending toward higher titers (Fig 6E). Interestingly, plasma responses in both cohorts were equally reactive to Man_9_V3 and aglycone V3 peptides, suggesting that V3/glycan bnAb site-specific antibody responses were low or nonexistent. We next determined whether the V3-directed antibodies could recognize native Env trimers. In fact, low but detectible binding titers were found to both JRFL and BG505 SOSIP Envs (Fig 6F), although the plasma did not neutralize these viruses (S3 Table). Tier 1 neutralization was also not observed, as expected, given that the immunodominant V3 crown epitope was not present (S4 Table). Taken together, these data demonstrate that this minimal peptide immunogen can be used to target the Env V3 epitope.

Antigen-specific B cell responses were assessed following vaccination by flow cytometry. Memory B cells were stained with Man_9_V3 and SOSIP Env probes to enumerate both vaccine-specific and SOSIP cross-reactive responses (Fig 6G). Vaccine-specific B cell responses were detected in most animals following three vaccinations, with responses trending higher in cohort 2 (average: 0.054% for cohort 1 versus 0.574% for cohort 2) (Fig 6H). In agreement with the serology, SOSIP cross-reactive B cells were found in most animals, albeit at a much lower level (range, 0.0013%–0.0994%).

### Star nanoparticles prime B cells displaying V3 bnAb characteristics

Because star nanoparticles displaying Man_9_V3 glycopeptides elicited B cells that could recognize SOSIP proteins, vaccine-specific mAbs were cloned to determine how this minimal immunogen was targeting the HIV Env. B cells were sorted based on their ability to bind both Man_9_V3 and aglycone V3 probes (S9A Fig), the Man_9_V3 probe only (S9C Fig), or Man_9_V3 and SOSIP probes (S9E Fig); genetic characteristics of the cloned mAbs are listed in S5 Table. As expected, mAbs cloned from B cells binding both glycosylated and non-glycosylated probes did not require the Man_9_ glycan for recognition (S9B Fig). Of seven mAbs that recognized V3 peptides, three bound weakly to JRFL SOSIP protein. From B cells that preferentially bound the Man_9_V3 over the aglycone V3 probe, two of nine mAbs were found to react only with Man_9_V3 and not aglycone peptides by ELISA, indicating at least partial glycan dependence (S9D Fig). Despite dependence on the Man_9_ glycans, these mAbs did not react with the JRFL SOSIP protein. From B cells that bound both the Man_9_V3 and SOSIP probes, it was notable that all mAbs were V3 specific, and they derived mostly from a single clone that did not show reactivity to the SOSIP protein by ELISA (S9F Fig). A less-prevalent secondary clone did, however, demonstrate weak binding. Finally, we assessed whether these mAbs reacted with the canonical “GDIR” motif targeted by many V3/glycan bnAbs. Using aglycone V3 peptides with alanine mutations in the GDIR motif, reactivity was measured for 12 mAbs from the aglycone+ (S10A and S10B Fig) and SOSIP+ (S10C and S10D Fig) sorted populations. Indeed, 3 of the 12 mAbs were dependent on the “R,” the “D,” or either “G”/“I” or “D”/“R” combinations of residues for binding. These data demonstrate that anti-V3 mAbs were elicited that could either bind to native Env trimers, could be dependent on mannose glycans, or target the “GDIR” motif. Taken together, the star nanoparticle vaccine primed an immune response focused to the V3 base that shares many of the characteristics of V3 bnAbs. However, given the overall weak reactivity with Env trimers, it is likely that additional immunogens will be needed to boost Env affinity and elicit neutralization.

### SOSIP boosting of V3-primed antibody responses

**Fig 7:**
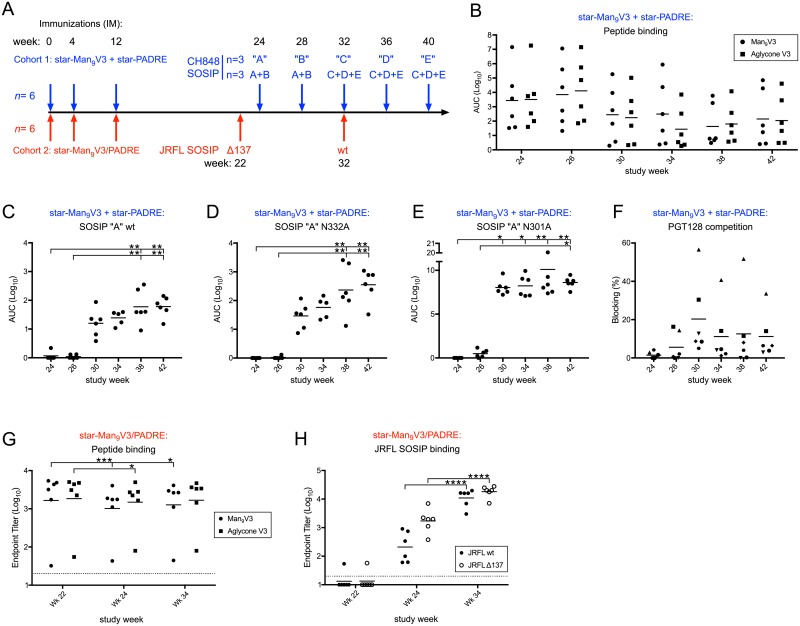
Immune responses following SOSIP trimer boosting of nanoparticle-primed NHP. (*A*) Immunization scheme for SOSIP boosts. (*B*) Serum binding titers measured from cohort 1 after each immunization to ELISA plates coated with Man_9_V3 or aglycone (non-glycosylated) V3 peptides. ELISA reactivity of cohort 1 sera to (*C*) SOSIP “A” wt, (*D*) SOSIP “A” N332A, and (*E*) SOSIP “A” N301A. (*F*) Competition from cohort 1 sera to PGT128 binding after each immunization. (*G*) Serum binding titers measured from cohort 2 after each immunization to ELISA plates coated with Man_9_V3 or aglycone (non-glycosylated) V3 peptides. (*H*) Serum binding titers measured from cohort 2 after each immunization to ELISA plates coated with wt or ΔN137 JRFL SOSIP. Data associated with this figure can be found in S1 Data. AUC, area under the curve; Man_9_V3, Env variable loop 3 oligomannose-9 glycopeptide; NHP, nonhuman primate; PADRE, pan DR epitope; V3, Env variable loop 3; wt, wild-type.

To attempt to boost the Env affinity and neutralization, two SOSIP boosting strategies were used. Cohort 1 was boosted with a series of five Env proteins derived from the human CH0848 donor, from whom the DH270 (a V3/glycan bnAb) lineage was isolated (Fig 7A and S6 Table) [34]. The recombinant CH0848 Env trimers were chimeric SOSIP proteins stabilized with A316W and E64K mutations and were antigenic for different members of the DH270 antibody lineage [15, 34, 49]. Of note, this SOSIP immunization regimen did not boost V3 peptide responses, as those binding titers continued to decline over time (Fig 7B). However, SOSIP binding titers were detectible after the second SOSIP immunization (Fig 7C, week 30) and increased thereafter. Interestingly, binding titers to SOSIP N332A (Fig 7D) and N301A (Fig 7E) proteins were as high or higher than titers to wild-type (wt) SOSIP, suggesting that the majority of the responses were not dependent on the glycans at those positions. Moreover, plasma was only modestly able to compete PGT128 binding to Env, blocking <20% in five of six animals, though one animal did attain 40%–60% blocking (Fig 7F). Significant tier 2 neutralization was not observed (S7 Table), although minimal tier 1 neutralization was detected (S4 Table). Taken together, these data suggest that while SOSIP boosting enhanced binding to Env, these SOSIP immunogens were not able to direct B cell lineages toward glycan reactivity and neutralization. Moreover, because the Man_9_V3 binding titers were not boosted, it is likely that the majority of the lineages primed by the star nanoparticles were not effectively engaged and matured.

Cohort 2 was boosted with two JRFL SOSIP immunogens, the first lacking the N137 glycan, which may promote maturation to the V3 region [50], followed by wt SOSIP (Fig 7A). Consistent with the cohort 1 data, a boost in serum responses to the V3 peptides was not observed following either SOSIP immunization (Fig 7G); however, autologous binding responses to JRFL Δ137 and wt SOSIPs were elicited after their respective immunizations (Fig 7H). Neutralization responses were not consistently observed, including to pseudoviruses produced in the presence of kifunensine (S7 Table). In conclusion, although immunogenic, neither set of SOSIP immunogens was able to drive the maturation of V3 lineages primed by the nanoparticles to induce significant neutralization.

## Discussion

Here, we report the design and characterization of a synthetic “star” nanoparticle vaccine capable of immunofocusing responses to an HIV-1 site of neutralization. This approach seeks to fully realize the potential of peptide minimal immunogens, which have been designed to replicate bnAb epitopes but are themselves too small to be immunogenic. Regarding HIV-1 minimal immunogens, most previous reports have relied on heterologous protein scaffolds for their structural stability and delivery [51–54]. Here, we have used a fully synthetic approach that enables the tuning of immunogenicity by varying properties such as particle size, immunogen density, and the co-delivery of T-cell helper peptides. In general, HIV-1 vaccine immunogenicity is often evaluated in various transgenic mouse models [55–58], which are useful for studying B cell lineage induction. However, we have evaluated our approach in NHPs, which are more challenging but may be a more relevant model. Compared with mice, NHP have similar TLR expression to humans, which is important for vaccine uptake and APC activation [59–61]. Additionally, because NHP are outbred, factors such as bnAb precursor frequency [23] and epitope immunodominance present additional and more realistic challenges [62].

Dendrimers are being evaluated in a number of clinical settings [63, 64], including as delivery vehicles for cancer therapeutics [65, 66], topical microbicides [67, 68], nucleic acids, and MRI contrast agents [69]. Similarly, HPMA-based polymers have been used as carriers for drugs [70–72], proteins [73], and gene delivery [74, 75]. However, to our knowledge this is the first report of dendrimer and HPMA conjugate nanoparticles being used to deliver immunogens for vaccination. Of note, the use of PHPMA-TLR7 conjugates was recently described using ovalbumin and the malaria circumsporozite protein (CSP) as immunogens [76]. That report focused on the use of polymers for immunogen and adjuvant co-delivery and APC targeting, but did not attempt immunogen multimerization. Given their favorable results with mannose APC targeting, it would be interesting to combine that concept with the multimerization that can be achieved by attaching PHPMA arms to dendrimer cores. Here, we have observed that immunogen multimerization directly correlates with improved immunogenicity (Fig 5A), likely the result of improved BCR cross-linking [77, 78].

In NHPs, star nanoparticles displaying Man_9_V3 and PADRE immunogens elicited high binding titers with weak affinity for SOSIP trimers. Two immunizations were required to achieve peak titers, which were not further improved by a third immunization. Because the goal of using the Man_9_V3 immunogen was to prime responses that could be further matured into bnAb responses, it was notable that we isolated B cell clones that had reactivity with SOSIP or were dependent on glycans for binding. Interestingly, Cai and colleagues have also recently reported serum glycan dependence based on a similar synthetic V3 minimal immunogen approach in rabbits [79]. Finally, three of the isolated mAb lineages targeted the GDIR motif, which is a common characteristic of V3 bnAbs [50, 80–82]. Thus, the B cell lineages being primed by this regimen share similar features with V3/glycan bnAbs despite being insufficient for neutralization.

One possible explanation for the lack of neutralization after nanoparticle priming is that antibodies to the Man_9_V3 immunogen had an angle of approach that was sterically or conformationally hindered in the context of the native trimer. The angle of approach is known to be a critical factor in bnAb activity [83–85], and V3 bnAbs are known to utilize several different approach angles [86, 87]. In fact, our mAbs with glycan dependence and GDIR reactivity had no detectible binding to SOSIP (S9 and S10 Figs), suggesting they may be approaching the epitope from a disfavored angle. This suggests a potential limitation to using minimal epitopes, because it may allow B cell recognition from too many angles. However, compared with the epitope diversity on full-length Env trimers, minimal immunogens still elicit a more focused response. Thus, the trimer immunogen is critical to boosting out antibody lineages with favorable angles of approach. In our studies, neither the CH0848 nor JRFL immunogens had detectible serum reactivity prior to boosting (Fig 7C and 7H); however, ELISA responses may not be detectable if the desired B cell lineages, i.e., those that bind SOSIP, were only a small fraction of the total response. More importantly, because the Man_9_- and aglycone-V3 peptide responses were not boosted (Fig 7B and 7G), this suggests that the SOSIP immunogens were not well matched to the primed responses. Future studies should use an ELISA-guided immunization boost strategy, as described by Escolano and colleagues [56].

Another potential solution to the challenges discussed above is to use star nanoparticles displaying Man_9_V3 glycopeptides as a boosting rather than priming immunogen. Such a study could benefit from SOSIP immunogens being designed to engage V3/glycan bnAb UCAs [34] or vaccine regimens shown to induce V3/glycan responses [82]. Following priming with native Env immunogens, which should only elicit antibodies with the correct angle of approach to the V3/glycan site, star-Man_9_V3 nanoparticles could be used to selectively boost out only the V3-directed B cell clones from the rest of the polyclonal responses primed by Env.

In conclusion, we evaluated a fully synthetic nanoparticle platform that allows for the correlation of biophysical parameters with vaccine immunogenicity. The data show that a dense array of V3 glycopeptide and PADRE minimal immunogens on the nanoparticle surface effectively engages B cells and other APCs and elicits high-titer antibodies in mice and NHPs, using a TLR7/8 agonist or other adjuvants. This approach can be used to focus antibody responses to a particular site of vulnerability on the HIV trimer. However, it remains to be determined how heterologous boosting regimens can be used to mature these responses to native Env trimers to elicit neutralization.

## Materials and methods

### Ethics statement

All animals were cared for in accordance with American Association for Accreditation of Laboratory Animal Care standards in accredited facilities. All animal procedures were performed according to protocols approved by the Institutional Animal Care and Use Committees of the National Institute of Allergy and Infectious Diseases, National Institutes of Health. Mouse studies were performed under animal study proposal protocol #VRC16-674; NHP studies were performed under animal study proposal protocol #VRC16-670.

### Synthesis of star-shaped copolymer

The star-shaped copolymer was prepared by attaching linear heterobifunctional polymers terminated with a TT group on one side and with propargyl (Pg) groups on the other side of the polymer chain to the surface amine groups on fifth-generation PAMAM dendrimers through acylation. Typically, a 5 wt. % methanol solution of the dendrimer (9.43 × 10^−7^ mol NH_2_ groups, 0.027 g) was added dropwise to a 10 wt. % solution of the polymer (6.03 × 10^−5^ mol, 0.828 g) in dry methanol and allowed to react overnight under argon atmosphere. The product was precipitated into diethyl ether and purified by membrane filtration (RC centrifugal filter units, MWCO 100 kDa) in PBS (4×, 0.15 mol∙L^−1^, pH 7.4) and in H_2_O (2×). The resulting star-shaped copolymer was isolated by lyophilization, yielding 0.352 g of white solid. *M*_w_ and *M*_w_/*M*_n_ of the star-shaped copolymer were 468.7 kDa and 1.09, respectively.

### Attachment of TLR-7/8a and peptides to the star-shaped copolymer

The TLR7/8 agonist adjuvant (2Bxy) was attached to the PAMAM core of the star-shaped copolymer in two steps using short heterobifunctional PEG linkers. First, the star-shaped copolymer (7.21 × 10^−5^ mol NH_2_ groups, 0.352 g) was dissolved in DMSO (10 wt. % solution), mixed with NHS-PEG_4_-DBCO (2.25 × 10^−5^ mol, 0.015 g) in 0.146 mL of DMSO and allowed to react 3 hours at r.t. Second, 2Bxy-N_3_ (2.25 × 10^−5^ mol, 0.011 g) was added to the reaction mixture and reacted for 3 hours at r.t. After that, the product was purified by the gravity SEC using Sephadex LH-20 in methanol and precipitated into diethyl ether, yielding 0.342 g of white solid. *M*_w_ and *M*_w_/*M*_n_ of the star-shaped copolymer bearing multiple TLR7/8 agonists were 476.1 kDa and 1.12, respectively.

All azide group–containing peptide immunogens were attached to the terminal Pg groups on the PHPMA grafts of the star-shaped copolymers via Cu^I^ catalyzed cycloaddition reaction in the presence of TBTA in DMSO/H2O (2:1) mixture. For example, equimolar amounts of star-shaped polymer (6.10 × 10^−7^ mol Pg groups, 10.0 mg), V3 peptide (6.10 × 10^−7^ mol, 2.1 mg), and TBTA (6.10 × 10^−7^ mol, 0.32 mg) were dissolved in DMSO (5 wt. % solution) and bubbled with argon. Then, the equimolar amount of CuBr (6.10 × 10^−7^ mol, 0.09 mg) was added to the reaction mixture; the solution was diluted with distilled water and allowed to react overnight at r.t. The resulting star-shaped copolymer/V3 peptide conjugate was mixed with 1 mL of 8-hydroxyquinoline (1 wt. % solution in methanol) and consecutively purified by the gravity SEC using Toyopearl HW-40F and Sephadex LH-20 in methanol. The methanol was evaporated to dryness, and the residue was dissolved with a defined volume of DMSO.

Additional information regarding polymer synthesis and characterization is provided in S1 Text. Biophysical properties of all nanoparticle constructs are detailed in S1 Table.

### Immunogens

The following peptide minimal immunogens were used. Prototype HIV V3 (unglycosylated), YNKRKRIHIGPGRAFYTTKNIIG [40]; HIV site of vulnerability Man_9_V3 (glycosylated), EINCTRPNNNTRPGEIIGDIRQAHCNISRA [29]; and PADRE, AKFVAAWTLKAAA. All peptides were synthesized with a C-terminal PEG spacer followed by an azido-Lys for nanoparticle attachment via “click chemistry.”

### HIV-1 Env SOSIP trimer production

Recombinant gp140 Envs were expressed as chimeric SOSIPs, where the gp120 of CH0848 was grafted onto the BG505 gp41 [88]. The trimers were stabilized with E64K and A316W mutations [15, 49]. Proteins were expressed in Freestyle293 cells (Invitrogen) using transient cotransfection of Env and furin DNA. Cell-free culture media was subjected to PGT151 antibody affinity and superdex200 size exclusion chromatography.

### Study animals

Female BALB/c mice, age <8 months, were obtained from The Jackson Laboratory (Bar Harbor, ME) and maintained at the Vaccine Research Center’s (VRC) Animal Care Facility (Bethesda, MD) under pathogen-free conditions. Twelve rhesus macaques of Chinese origin were divided into two cohorts of 6 and housed with the NIH Department of Veterinary Resources.

### Mouse route and dosing

For mice, vaccine doses consisted of 5 μg of V3 and 5 μg of PADRE peptides, formulated as soluble peptides or immobilized on star nanoparticles and then admixed with various adjuvants in a 50-μL volume. The TLR7/8 agonist was dosed at 5 nmoles; Addavax at 25 μL; and pIC:LC, Alhydrogel, and Adju-Phos at 50 μg. Immunizations were given at 0 and 3 weeks, with serum sampling at day 10 and week 5. Immunizations were given SC (footpad), IM (quadriceps), or IV (tail vein), as indicated. Because a limited number of TLR7/8 agonist molecules could be attached to the dendrimer core, the TLR7/8 dose in the route of immunization study was approximately 2 nmoles.

### NHP route and dosing

All NHPs were immunized IM in the quadriceps with star nanoparticles at 0, 4, and 12 weeks. Cohort 1 was boosted with Env at weeks 24, 28, 32, 36, and 40; cohort 2 was boosted with Env at weeks 22 and 32. Peak sampling was taken 2 weeks after each immunization. Immunogen dosing is described in S1 Table. For cohort 1, the dose of the TLR7/8 agonist adjuvant was 17 nmoles as a safety precaution for the first immunization. For all subsequent doses in cohort 1 and all doses in cohort 2, 200 nmoles was used. For all SOSIP Env boost immunizations, 100 μg of protein was admixed with 1 mg polyIC:LC (Hiltonol; Oncovir, Washington, DC) in a 1-mL volume, which was split between both quadriceps. Cohort 1 was subdivided such that three NHPs received 100 μg of only one of five CH0848-derived envelopes (S6 Table) given in series, while three NHPs received a mixture of two or three envelopes (Fig 7a) at 50 μg or 33.3 μg each, respectively, for a total of 100 μg. For cohort 2, all six NHPs received 100 μg of JRFL N137A (week 22) and JRFL wt SOSIP protein (week 32).

### Statistics

Statistical comparisons were performed using Prism 7.0 software (GraphPad). For analyses between multiple vaccine groups, a Kruskal-Wallis test with Dunn’s correction was used, with *p*-values indicated by asterisks. For analyses between groups over time, a two-way ANOVA with Bonferroni correction was used, with comparisons between vaccine groups at each time point depicted by a single asterisk for any *p*-value less than 0.05. For analyses between only two groups, a Wilcoxon matched-pairs signed-rank test was used.

The characterization of vaccine responses was performed as described previously [89–93], and is detailed in S1 Text.

## Supporting information

S1 Table Properties of star nanoparticles used in this study. All constructs are based on a G5 PAMAM core with 10-kD HPMA polymer arms. The use of each construct in this study is referenced by the figure legend (first column). G5, fifth-generation; HPMA, *N*-[(2-hydroxypropyl)methacrylamide]; Mn, number average molecular weight; Mw, weight average molecular weight; n/a, not applicable; N.d., not determined; PAMAM, poly(amidoamine); Rg, radius of gyration; Rh, hydrodynamic radius.
(XLSX)

S2 Table NHP dosing of star nanoparticles for each immunization. Note that a lower dose was used in the first immunization for the star-Man_9_V3 + star-PADRE regimen (cohort 1), which was done as a safety precaution. Subsequently, nanoparticles were given the higher 27 nmole dose for all immunizations. Also note that in order to keep constant the Man_9_V3 peptide dose, the PADRE dose was necessarily lower in the star-Man_9_V3/PADRE regimen because of limits on the number of PADRE peptides that could be attached to the nanoparticles. Man_9_V3, Env variable loop 3 oligomannose-9 glycopeptide; NHP, nonhuman primate; PADRE, pan DR epitope.
(XLSX)

S3 Table NHP plasma neutralization following priming immunizations with star-Man_9_V3 + star-PADRE nanoparticles at weeks 0, 4, and 12 (cohort 1). Values represent reciprocal serum dilution required to achieve 50% (ID50) neutralization. Plasma was tested after 2 (week 6) or 3 (week 14) immunizations. ID50, infectious dose 50; Man_9_V3, Env variable loop 3 oligomannose-9 glycopeptide; NHP, nonhuman primate; PADRE, pan DR epitope.
(XLSX)

S4 Table NHP plasma tier 1 neutralization from cohort 1 following priming immunizations with star-Man_9_V3 + star-PADRE nanoparticles (week 14) or boosting with SOSIP immunogens (week 42). Values represent the reciprocal serum dilution required to achieve 50% (ID50) neutralization of the tier 1 viruses SF162 and MW965. ID50, infectious dose 50; Man_9_V3, Env variable loop 3 oligomannose-9 glycopeptide; NHP, nonhuman primate; PADRE, pan DR epitope.
(XLSX)

S5 Table Heavy chain genetic characteristics of NHP monoclonal antibodies isolated following immunization. Aglycone V3+ and Man_9_V3+ mAbs were isolated from animals from cohort 1; Man_9_V3+/SOSIP+ mAbs were isolated from an animal in cohort 2. Sort phenotype refers to the probe binding phenotype, as depicted in Fig 6a, 6c and 6e; mAb IDs also match with those listed in Fig 6. Multiple gene listings indicate the sequence could map to these genes with similar identity. mAb, monoclonal antibody; Man_9_V3, Env variable loop 3 oligomannose-9 glycopeptide; NHP, nonhuman primate; V3, Env variable loop 3.
(XLSX)

S6 Table SOSIP Env immunogens used for boosting immunizations in cohort 1. Env clones were isolated from the CH0848 donor, as previously described in Bonsignori and colleagues [34]. Staged induction of HIV-1 glycan-dependent broadly neutralizing antibodies. Env, HIV envelope protein.
(XLSX)

S7 Table NHP plasma neutralization following SOSIP boosting immunizations in animals primed with star-Man_9_V3 + star-PADRE nanoparticles (weeks 24, 28, 32, 36, and 40; cohort 1, top) or star-Man_9_V3/PADRE nanoparticles (weeks 22 and 32; cohort 2, bottom). Cohort 1 neutralization was measured at weeks 0, 4, and 12. Cohort 2 neutralization was measured at weeks 0, 4, and 12. Values represent reciprocal serum dilution required to achieve 50% (ID50) neutralization; green shading indicates values above the assay limit of detection; blank spaces indicate samples were not tested. ID50, infectious dose 50; Man_9_V3, Env variable loop 3 oligomannose-9 glycopeptide; NHP, nonhuman primate; PADRE, pan DR epitope.
(XLSX)

S1 Fig Immunogenicity of minimal peptide immunogens using different adjuvant formulations. A dose of 5 μg of V3 and 5 μg of PADRE peptides were admixed with 25 μL (2% oil [final]) SAS, 25 μL AddaVax, or 50 μg pIC:LC or conjugated to star nanoparticles bearing a TLR7/8 agonist. In a separate study, V3 and PADRE peptides were admixed with 50 μg Alhydrogel or 50 μg Adju-Phos and compared with their conjugation to star-TLR7/8 nanoparticles. Serum end point titers were measured after two homologous immunizations by ELISA. Bars indicate group geometric means; horizontal lines indicate ELISA assay limit of detection. **p < 0.01 by the Kruskal-Wallis test. Data associated with this figure can be found in S1 Data. PADRE, pan DR epitope; pIC:LC, polyinosinic-polycytidylic acid; SAS, Sigma Adjuvant System; TLR7/8, Toll-like receptor 7/8; V3, Env variable loop 3.
(EPS)

S2 Fig SEC analysis of the linear heterotelechelic polymer TT-P(HPMA)-Pg (black lines) and the star copolymer P(AMAM)-*g*-P(HPMA)-Pg (red lines). Solid lines represent an LS detector response; dashed lines represent a differential RI detector response. LS, light scattering; RI, refractive index; SEC, size exclusion chromatography.
(TIFF)

S3 Fig Scheme of conjugation of a small molecule adjuvant (TLR7/8 agonist) to the PAMAM dendrimer: (i) modification of free primary amino groups on the PAMAM surface with the DBCO-PEG4-NHS ester linker through the amide bond formation and (ii) conjugation of azide group–containing TLR7/8 agonist to the DBCO groups on the PAMAM surface through the triazole bond formation. DBCO, dibenzocyclooctyne; NHS, N-hydroxysuccinimide; PAMAM, poly(amidoamine); PEG, polyethylene glycol; TLR7/8, Toll-like receptor 7/8.
(TIFF)

S4 Fig Flow cytometry gating trees. (*A*) Lymph node–resident APCs. B cells are defined as CD19^+^, B220^+^; plasmacytoid DCs: CD19^−^, B220^+^; neutrophils: Ly-6G^+^; monocyte-derived DCs: CD11c^+^, F4/80^+^, Cy-6C^+^; cDC1 (resident) DCs: CD11c^+^, F4/80^−^, CD8a^+^, CD11b^−^; cDC1 (migratory) DCs: CD11c^+^, F4/80^−^, CD8a^−^, CD11b^−^; cDC2 DCs: CD11c^+^, F4/80^−^, CD8a^−^, CD11b^+^. (*B*) Cytokine-producing splenocytes. T cells were gated from based CD3 and CD4 or CD8 expression, followed by gating on IL-2, TNFα, and IFN-γ expression. A PMA/ionomycin stimulation is shown as a positive control for clarity. APC, antigen-presenting cell; CD, cluster of differentiation; cDC, conventional dendritic cell; DC, dendritic cell; IL-2, interleukin 2; IFN-γ, interferon gamma; PMA, phosphomolybdic acid; TNFα, tumor necrosis factor alpha.
(EPS)

S5 Fig Histocytometric analysis of vaccine uptake in draining LNs. Confocal images were converted into flow cytometry data by identifying single cells at given y- and x-coordinates and analyzing the expression levels of various APC markers. The vaccine+ gate was set conservatively to avoid B cells, which were found to nonspecifically take up a low level of the vaccine (main Fig 3). Subset gating is as follows: subcapsular macrophages, CD169^+^ CD11b^+^; undefined CD169^+^ cells, CD169^+^ CD11b^−^; all other monocytes, CD169^−^ CD11b^+^; cDC2 or monocyte-derived DCs, CD11c^+^ CD11b^+^; resident DCs, MHCII^lo^ CD11c^hi^; migratory DCs, MHCII^hi^ CD11c^+^. Populations of interest were then replotted by their y- and x-coordinates and color coded. Note that for simplicity, histocytometry plotting by x- and y-coordinates is limited such that populations listed in the legend first supersede lower-listed populations in cases of marker ambiguity. For example, CD169^+^ CD11b^+^ cells may also be CD11c^+^, but because cyan cells are listed above red cells, red cells are CD169^−^ by definition. Similarly, yellow cells mark macrophages that are CD11c^−^. Finally, the plotted resident and migratory DC subsets are those that are CD11b^−^, even though MHCII^lo^ CD11c^hi^ cells are often also CD11b^+^ (such CD11c CD11b–expressing cells are plotted as red). APC, antigen-presenting cell; CD, cluster of differentiation; DC, dendritic cell; LN, lymph node; MHCII, major histocompatibility complex class II.
(EPS)

S6 Fig Subcapsular macrophage disruption following vaccination. LNs were harvested 4 hours after footpad immunization, with star nanoparticles bearing Ax647-labeled V3 peptides, unlabeled nanoparticles bearing PADRE peptides, and a soluble TLR7/8 agonist adjuvant (*A*), or without any adjuvant (*B*). Confocal microscopy was used to visualize vaccine nanoparticles and CD169+ SCS macrophages in the vaccinated draining (ipsilateral) or unvaccinated (contralateral) LNs. Scale bars indicate 100 μm, except for adjuvanted example 2, 150 μm. Ax647, Alexa Fluor 647; LN, lymph node; PADRE, pan DR epitope; SCS, subcapsular sinus; TLR7/8, Toll-like receptor 7/8; V3, Env variable loop 3.
(EPS)

S7 Fig Immunogenicity of minimal peptide immunogens displayed on linear or star polymer formulations. A dose of 5 μg of unformulated V3 peptides was administered as a mixture with a polymeric TLR7/8 agonist or after conjugation to a linear PHPMA polymer at X or XX peptides per polymer, and compared with V3 peptides conjugated to star nanoparticles bearing a TLR7/8 agonist. Serum end point titers were measured after two homologous immunizations by ELISA. Bars indicate group geometric means; horizontal lines indicate ELISA assay limit of detection. **p < 0.01 by the Kruskal-Wallis test. Data associated with this figure can be found in S1 Data. PHPMA, poly[N-(2-hydroxypropyl) methacrylamide]; TLR7/8, Toll-like receptor 7/8; V3, Env variable loop 3.
(EPS)

S8 Fig T-cell responses following vaccination with star nanoparticles bearing V3 and PADRE peptides. (*A*) Mice were immunized with soluble V3 peptides, with or without star nanoparticles bearing PADRE peptides, with star nanoparticles bearing V3 or PADRE peptides on separate particles, or with star nanoparticles bearing both V3 and PADRE peptides on the same particle; all vaccines were adjuvanted by admixing with soluble TLR7/8a. After three homologous immunizations, splenocytes were harvested and restimulated ex vivo with V3 or PADRE peptides. Antigen-specific T-cell responses were measured after stimulation by intracellular cytokine staining and flow cytometry. (*B*) Star nanoparticles bearing TLR7/8 agonists, V3, and PADRE peptides were administered IM, SC, or IV. After two homologous immunizations, splenocytes were harvested and restimulated ex vivo with PADRE peptides. Antigen-specific T-cell responses were measured after stimulation by intracellular cytokine staining and flow cytometry. Data are representative of at least two independent experiments. *p < 0.05; **p < 0.01; ***p < 0.001; ****p < 0.0001; n.s., not significant by the Kruskal-Wallis test. Data associated with this figure can be found in S1 Data. IM, intramuscularly; IV, intravenously; n.s., not significant; PADRE, pan DR epitope; SC, subcutaneously; TLR7/8a, Toll-like receptor 7/8; V3, Env variable loop 3.
(EPS)

S9 Fig Characterization of NHP monoclonal antibodies elicited by star nanoparticles bearing the Man_9_V3 immunogen. (*A*,*C*,*E*) B cells were sorted based on their ability to bind Man_9_V3, aglycone V3, and SOSIP probes. (*B*,*D*,*F*) Antibodies were screened for binding by ELISA for reactivity to Man_9_V3, aglycone V3, and JRFL SOSIP Env. (*A*,*B*) mAbs cloned from B cells binding both Man_9_V3 and aglycone V3 probes. Stars indicate mAbs that reacted with SOSIP. (*C*,*D*) mAbs cloned from B cells binding the Man_9_V3 but not the aglycone V3 probe. Stars indicate mAbs that were specific for Man_9_V3. (*E*,*F*) mAbs cloned from B cells binding both the Man_9_V3 and SOSIP probes. Stars indicate mAbs that reacted with SOSIP. Horizontal lines indicate the ELISA assay limit of detection; boxes indicate the number of mAbs that were positive in each ELISA screen. Data associated with this figure can be found in S1 Data. Env, HIV envelope protein; mAb, monoclonal antibody; Man_9_V3, Env variable loop 3 oligomannose-9 glycopeptide; NHP, nonhuman primate; V3, Env variable loop 3.
(EPS)

S10 Fig Characterization of NHP mAb binding to the GDIR motif common to human anti-V3 bnAbs. (*A*) Monoclonal antibodies that were cloned based on binding to both the Man_9_V3 and aglycone V3 probes were screened for binding to V3 peptides containing mutations in the GDIR motif (*B*). mAb 03 displays sensitivity to changes at the “R” residue, while mAb 34 displays sensitivity to changes at the “D” and “G” or “I” residues. (*C*) mAbs that were cloned based on binding to both the Man_9_V3 and SOSIP Env probes were screened for binding to V3 peptides containing mutations in the GDIR motif (*D*). mAb 04 displays sensitivity to changes at the “R” and “G” or “I” residues. Data associated with this figure can be found in S1 Data. bnAb, broadly neutralizing antibody; Env, HIV envelope protein; mAb, monoclonal antibody; Man_9_V3, Env variable loop 3 oligomannose-9 glycopeptide; NHP, nonhuman primate; V3, Env variable loop 3.
(EPS)

S1 Data Raw data values that correspond to the graphs displayed in the main and supplemental figures. (XLSX)

S1 Text Supporting materials and methods. (DOCX)

## Abbreviations

APC: antigen-presenting cell
AUC: area under the curve
Ax647: Alexa Fluor 647
BCR: B cell receptor
BLI: biolayer interferometry
bnAb: broadly neutralizing antibody
CD: cluster of differentiation
CD4bs: CD4 binding site
cDC: conventional dendritic cell
CSP: circumsporozite protein
DC: dendritic cell
Env: HIV envelope protein
eOD: Env outer domain
G5: fifth-generation
GC: germinal center
GlcNAc: *N*-acetylglucosamine
HPMA: *N*-[(2-hydroxypropyl)methacrylamide]
IgG: immunoglobulin G
IM: intramuscularly
IV: intravenously
LN: lymph node
mAb: monoclonal antibody
Man_9_V3: Env variable loop 3 oligomannose-9 glycopeptide
MFI: median fluorescence intensity
MHCII: major histocompatibility complex class II
NHP: nonhuman primate
PADRE: pan DR epitope
PAMAM: poly(amidoamine)
pDC: plasmacytoid dendritic cell
Pg: propargyl
PHPMA: poly[N-(2-hydroxypropyl) methacrylamide]
polyIC:LC: polyinosinic-polycytidylic acid
SC: subcutaneously
SCS: subcapsular sinus
SHM: somatic hypermutation
TLR: Toll-like receptor
TT: thiazolidine-2-thione
UCA: unmutated common ancestor
V1: Env variable loop 1
V2: Env variable loop 2
V3: Env variable loop 3
VRC: Vaccine Research Center
wt: wild-type
