# Supplementary figures and images for: Star nanoparticles delivering HIV-1 peptide minimal immunogens elicit near-native envelope antibody responses in nonhuman primates
Source: PLoS Biol. 2019 Jun 17;17(6):e3000328. doi: 10.1371/journal.pbio.3000328 (PMC6597128; doi:10.1371/journal.pbio.3000328)

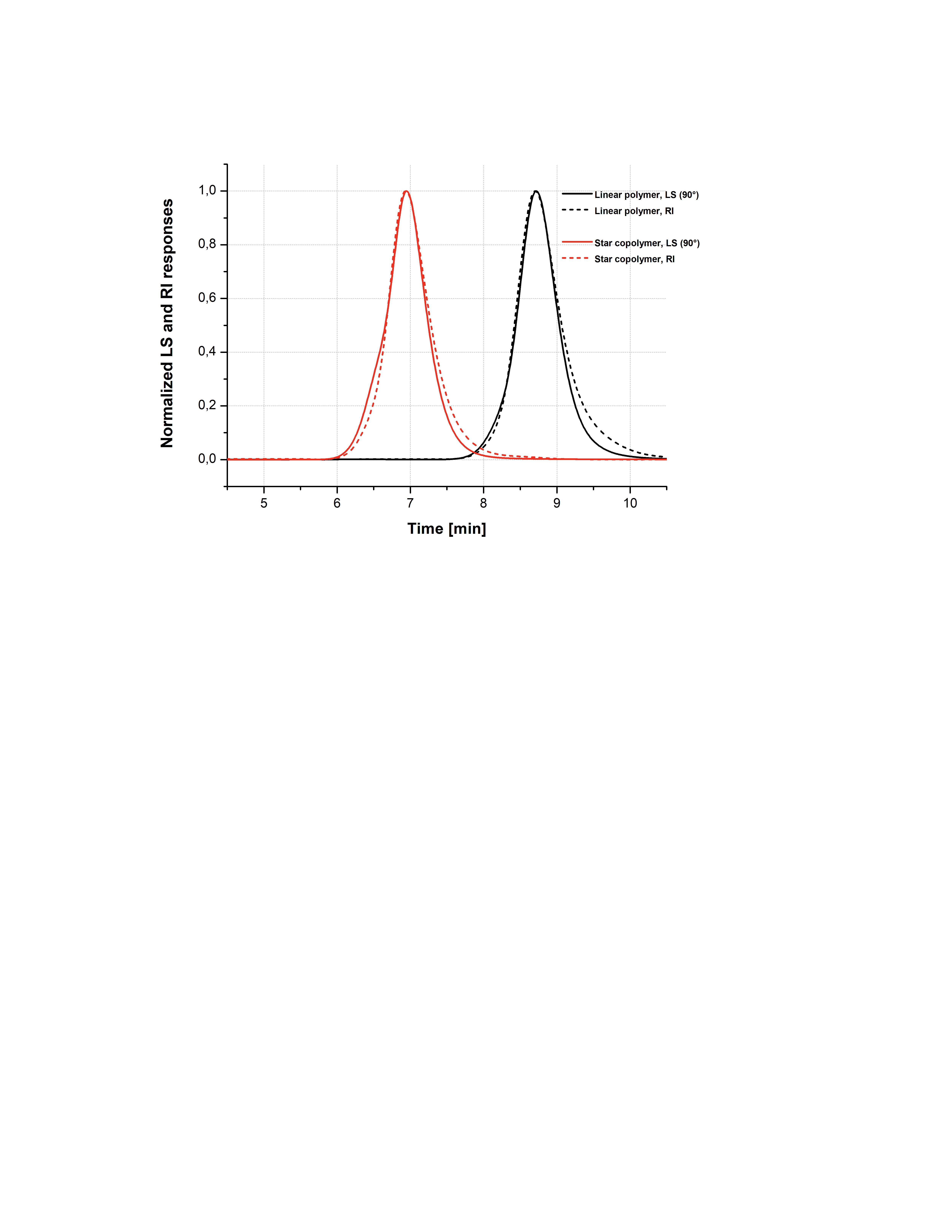

Supplement: S2 Fig — Solid lines represent an LS detector response; dashed lines represent a differential RI detector response. LS, light scattering; RI, refractive index; SEC, size exclusion chromatography. (TIFF) [file pbio.3000328.s009.tiff]

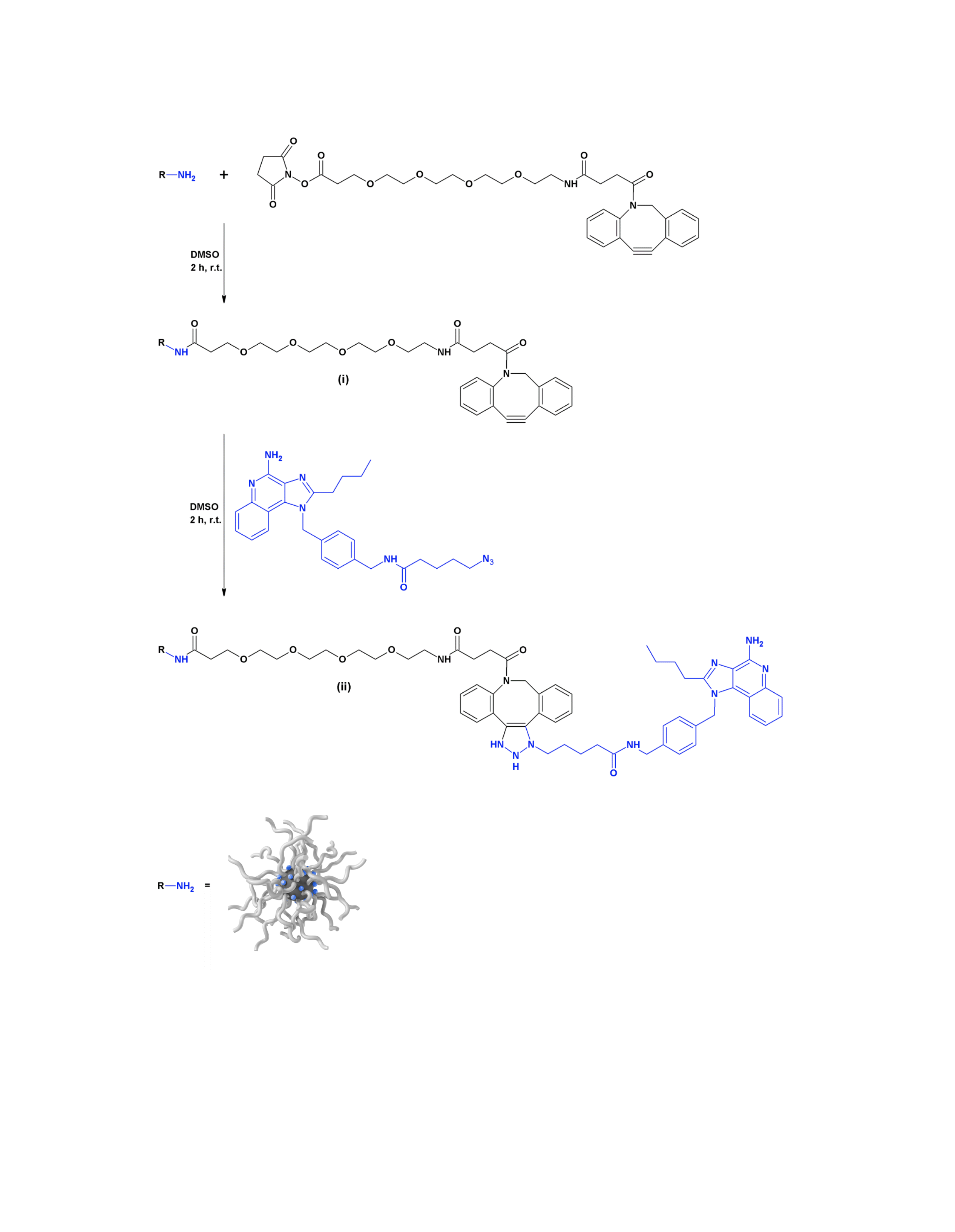

Supplement: S3 Fig — DBCO, dibenzocyclooctyne; NHS, N-hydroxysuccinimide; PAMAM, poly(amidoamine); PEG, polyethylene glycol; TLR7/8, Toll-like receptor 7/8. (TIFF) [file pbio.3000328.s010.tiff]
