# Supplementary material for: Star nanoparticles delivering HIV-1 peptide minimal immunogens elicit near-native envelope antibody responses in nonhuman primates
Source: PLoS Biol. 2019 Jun 17;17(6):e3000328. doi: 10.1371/journal.pbio.3000328 (PMC6597128; doi:10.1371/journal.pbio.3000328)
Supplement: S1 Text — (DOCX) [file pbio.3000328.s019.docx]

**Supporting Materials and Methods:**

*Nanoparticle synthesis and characterization*

**Chemicals.** (RS)-1-Aminopropan-2-ol, L-ascorbic acid; 4,4′-azobis(4-cyanovaleric acid) (ACVA); bis(thiobenzoyl) disulfide; copper(I) bromide; dicyclohexylcarbodiimide (DCC); *N,N′*-diisopropylethylamine (DIPEA); *N*-(3-dimethylaminopropyl)-*N′*-ethylcarbodiimide hydrochloride (EDC); 4-(dimethylamino)pyridine (DMAP); 8-hydroxyquinoline; methacryloyl chloride; poly(amidoamine) dendrimer, ethylenediamine core, generation 5.0 (PAMAM); propargylamine; thiazolidine-2-thione; tris[(1-benzyl-1H-1,2,3-triazol-4-yl)methyl]amine (TBTA) were purchased from Sigma-Aldrich, Czech Republic. Dibenzocyclooctyne-PEG4-N-hydroxysuccinimidyl ester (DBCO-PEG4-NHS) was obtained from Click Chemistry Tools, USA. All solvents used in this work were of high-purity grade with extremely low water levels purchased from VWR, Czech Republic.

**Synthesis of monomers.** *N*-(2-Hydroxypropyl)methacrylamide (HPMA) was synthesized by reacting methacryloyl chloride with (RS)-1-aminopropan-2-ol in dichloromethane in the presence of sodium carbonate as described in (1).

**Synthesis of initiators and RAFT agent.** 2-[1-Cyano-1-methyl-4-oxo-4-(2-thioxothiazolidin-3-yl)butylazo]-2-methyl-5-oxo-5-(2-thioxothiazolidin-3-yl)pentanenitrile (ACVA-(TT)2) was prepared by the reaction of ACVA with thiazolidine-2-thione in tetrahydrofuran in the presence of DCC and DMAP (2).

4-Cyano-4-(1-cyano-3-ethynylcarbamoyl-1-methylpropylazo)-*N*-ethynyl-4-methylbutyramide (ACVA-(Pg)_2_) was prepared by reacting ACVA with propargylamine in dichloromethane the presence of EDC and DMAP (2). Dithiobenzoic acid 1-cyano-1-methyl-4-oxo-4-(2-thioxothiazolidin-3-yl)butyl ester (CTA-TT) was synthesized by the reaction of ACVA-(TT)_2_ with bis(thiobenzoyl) disulfide in ethyl acetate at 80°C as described in (2).

**Synthesis of linear co-polymers**. Statistical linear co-polymer with propargyl functional groups was prepared by RAFT co-polymerization of N-(2-hydroxypropyl)methacrylamide (HPMA) with 3-(3-methacryloylamidopropanoyl)thiazolidin-2-thion (Ma-βAla-TT) in the presence of 2-cyano-2-propyl benzodithioate (CPB) as a chain-trasfer agent and 2,2′-azobis(2-methylpropionitrile) (AIBN) as an initiator followed by a post-polymerization modification of synthesized co-polymer with propargylamine. Briefly, a mixture of HPMA (500.0 mg, 3.49 mmol), Ma-βAla-TT (100.2 mg, 0.39 mmol), CPB (3.2 mg, 14.6 μmol) and AIBN (1.2 mg, 7.3 μmol) was dissolved in 4.3 mL of tert-butanol/DMSO (9:1), thoroughly bubbled with argon and polymerized at 70 °C for 16 h. Afterwards, the polymerization mixture was precipitated to aceton/diethylether (3:1) and re-precipitated from methanol to aceton/diethylether (3:1) yielding 435.0 mg of orange colored powder. To remove dithiobenzoate (DTB) end group, a mixture of co-polymer and AIBN (61.2 mg, 0.37 mmol) was dissolved in 4.4 mL of DMSO, bubbled with argon and heated to 80 °C for 2 h. The co-polymer was isolated by precipitation to aceton/diethylether (3:1) followed by re-precipitation from methanol to aceton/diethylether (3:1) yielding 425.0 mg of yellow powder. Next, propargylamine (35.2 μL, 0.55 mmol) was added to the solution of the co-polymer in 4.3 mL of DMSO and the reaction mixture was stirred at r.t. for 2 h. The co-polymer was precipitated to diethyl ether, re-dissolved in methanol and purified by gel filtration using a Sephadex LH-20 in methanol. The resulting co-polymer was isolated by precipitation to diethyl ether yielding 412.3 mg of white powder. The number-average molecular weight and polydispersity index of the p[(HPMA)-co-(Ma- βAla-Pg)] co-polymer determined by SEC were 33 kDa and 1.02, respectively. The molar content of propargyl (Pg) reactive groups determined by 1H-NMR was 8.9 mol. %.

**Syntheses of linear co-polymer-V3 peptide conjugate**. Azide group-terminated V3 peptide antigen (YNKRKRIHIGPGRAFYTTKNIIG-PEG12-N3) was conjugated to the p[(HPMA)-co-(Ma- βAla-Pg)] linear co-polymer by the CuI-catalyzed Huisgen cycloaddition in the presence of tris[(1-benzyl-1H-1,2,3-triazol-4-yl)methyl]amine (TBTA). Briefly, a mixture of the linear co-polymer (12.2 mg) and sodium ascorbate (1.83 mg, 9.2 μmol) was dissolved in 1.1 mL of deionized water and thoroughly bubbled with argon. To this solution, V3 peptide (16.0 mg, 4.6 μmol) and TBTA (2.46 mg,  4.6 μmol) in 1.1 mL of DMSO was added and the mixture was thoroughly bubbled with argon. Finally, CuSO4 · 5H2O (2.46 mg, 4.6 μmol) was added and the reaction mixture was stirred overnight at r.t. Afterwards, the solution was diluted with 8-hydroxyquinoline (1.0 M, 2.2 mL) and loaded to a Sephadex LH-20 column in methanol. Methanol was evaporated to yield 9.8 mg of the resulting conjugate. Then, the conjugate was dissolved in DMSO to a final concentration of 20 mg/mL and preserved at -20 °C. The number-average molecular weight and polydispersity index of the p[(HPMA)-co-(Ma- βAla-V3)] co-polymer determined by SEC were 73 kDa and 1.1, respectively. The molar content of V3 peptide determined by amino acid analysis was 5.1 mol. %, which corresponds to ~11.5 of V3 peptide units per polymer chain. The same strategy was used to generate the conjugate with ~half molar content of V3 peptide. In this case, the number-average molecular weight and polydispersity index were 48 kDa and 1.1, respectively. The molar content of V3 peptide was 1.9 mol. %, which corresponds to ~4.3 of V3 peptide units per polymer chain.

**Synthesis of Toll-like receptor 7/8 agonists (TLR7/8a).** 1-(4-{[(5-Azidopentanoyl)amino]methyl}phenyl))-2-butyl-1H-imidazo[4,5-c]quinolin-4-amine (2Bxy-N3) was prepared by the multistep synthesis starting from quinolone -2,4-diol as described elsewhere (3, 4).

**Synthesis of heterobifunctional linear polymer.** Linear polymer of approximately 14 kDa was synthesized via radical addition-fragmentation chain transfer polymerization (RAFT). Typically, HPMA (0.014 mol, 2.000 g), CTA-TT (1.03e^-4^ mol, 0.040 g) and ACVA-(TT)_2_ (5.15e^-5^ mol, 0.025 g) were dissolved in 0.9 mol∙L^-1^ mixture of *tert*-butanol/DMSO (9:1), bubbled with argon, sealed into glass ampoule and polymerized at 70°C for 16 hours. Afterward, the polymer was precipitated into acetone/diethyl ether (3:1) and re-precipitated from methanol yielding 0.880 g of pink solid. *M*_w_ and *M*_w_/*M*_n_ of the polymer were 13.7 kDa and 1.04, respectively. The polymer dithiobenzoate (DTB) end groups were further converted to the propargyl groups by the homolytic reaction with an excess of ACVA-(Pg)_2_. Typically, the polymer (6.42e^-5^ mol ~DTB groups, 0.880 g) and ACVA-(Pg)_2_ (1.28e^-3^ mol, 0.455 g) were dissolved in DMSO (10 wt% solution), sealed in a glass ampoule and heated at 80°C for 2 hours. The polymer was precipitated into acetone/diethyl ether (3:1) and re-precipitated from methanol yielding 0.828 g of pale yellow solid. *M*_w_ and *M*_w_/*M*_n_ of the polymer were 13.9 kDa and 1.08, respectively. The content of TT end groups was 7.19e^-5^ mol/g.

**UV-VIS spectrophotometry.** The spectrophotometric analyses of the polymers were carried out in quartz glass cuvettes on a UV-VIS spectrophotometer Specord Plus (Analytik Jena, Germany). The content of dithiobenzoate (DTB) end groups in the linear polymers was determined at 302 nm in methanol using the molar absorption coefficient 12,100 L/mol·cm. The content of carbonylthiazolidine-2-thione (TT) reactive end groups in the linear polymers was determined at 305 nm using the molar absorption coefficient 10,300 L/mol·cm. The determination of the TLR7/8a in the star-shaped co-polymers was performed at 325 nm in methanol using the molar absorption coefficient 5,012 L/mol·cm.

**Size-exclusion chromatography (SEC).** The molecular weights, molecular weights distributions and gyration radii of the polymers were determined by SEC on a HPLC system (Shimadzu VP, Japan), equipped with internal UV–VIS photodiode array detector, and external differential refractive index and multiangle light scattering detectors (Wyatt Technologies, USA). The TSK-Gel SuperAW3000 and SuperAW4000 columns (6.0 × 150 mm, Tosoh Bioscience, Japan) connected in series and 80% methanol / 20% sodium acetate buffer (0.3 M, pH 6.5) mixture as a mobile phase (flow rate 0.6 mL/min) were used in all experiments. A method based on the known total injected mass with an assumption of 100% recovery was used for the calculation of the refractive index increments (dn/dc) needed for the molecular weights determination from light scattering data.

**High-performance liquid chromatography (HPLC).** The purity of all low-molecular-weight compounds synthesized in this study as well as the course of the modification of the star-shaped polymers with the TLR7/8a and/or peptides were monitored by the liquid chromatography on a HPLC system (Shimadzu VP, Japan) equipped with UV-VIS photodiode array detector using a reversed-phase column Chromolith RP18-e (4.6 × 100 mm, Merck, Germany), with a linear gradient (0 − 100%) of water/acetonitrile mixture containing 0.1% TFA at a flow rate 2.5 mL/min.

**Dynamic light scattering (DLS).** The hydrodynamic diameters (*D*_H_) of the polymers were determined by the DLS technique at a scattering angle 173° using a Nano-ZS instrument (Malvern Instruments, UK) equipped with a 4 mW, 633 nm laser. The measurements were performed in PBS solution (0.15 mM, pH 7.4) at 37°C. For the evaluation of the dynamic light scattering data, the DTS (Nano) program was used. The resulting *D*_H_ values were arithmetic means of at least ten independent measurements.

**Electron microscopy.** Samples were diluted to ~0.01 mg/mL and adsorbed to a freshly glow-discharged carbon-film grid for 15 s. After three washes, the adsorbed nanoparticles were stained with 0.7% uranyl formate. Images were collected on an FEI Tecnai T20 electron microscope equipped with a 2k x 2k Eagle CCD camera at a nominal magnification of 100,000 (pixel size: 0.22 nm).

**Biolayer interferometry (BLI).** BLI was used to measure the antigenicity of immunogens and vaccine formulations. On an Octet Red384 instrument (fortéBio) monoclonal antibodies were immobilized using anti-human F_C_ capture biosensors (fortéBio) as the ligand, for association with the vaccine formulations as the analyte. To measure the antigenicity of the Man_9_V3 or aglyconeV3 immunogens for PGT128 and VRC41 (Fig 5B), biotinylated forms of the V3 peptides were immobilized onto streptavidin sensors as the ligand, then associated with titrations (267-33 nM) of the IgG in solution as the analyte. Analytes were plated in solid black tilt-well 96-well plates (Geiger Bio-One); assays were performed with agitation at 30°C. Ligand loading was performed for 300s, followed by a 60s baseline in buffer (PBS+1% BSA). Association with the vaccine was carried out for 300s, followed by a dissociate step in buffer for 600 s.

*Vaccine immunological characterization*

**Animal sampling.** NHP sera and PMBC sampling was performed as previously reported (5).

**In vivo mouse imaging.** Vaccine pharmacokinetics were measured by live animal imaging. Animals were first anesthetized with isoflurane gas. Animals were then imaged in parallel with x-ray and fluorescence scans using an In Vivo Xtreme imaging system (Bruker), during which they were supplied isoflurane through a muzzle nozzle. The Ax647 label was imaged by excitation at 650 nm for 0.915 s; emission was read at 700 nm. Data were analyzed using Molecular Imaging Software “MI” (Bruker). Fluorescence was quantified by drawing a region of interest (ROI) gate over the left foot pad and applying the same gate to all images. The photons/s/mm^2^ within the ROI were then measured by the software and net signal calculated for each mouse at each time point. Images for figures were generated by setting the same minimum and maximum fluorescence intensities for each image and overlaying the fluorescence on the X-ray.

*Lymph node analyses*

**Flow cytometry.** Popliteal LNs were harvested after vaccination and mechanically disrupted in PBS + 1 μg/mL collaginase D and 100 U/mL DNAseI, then centrifuged through a 96 well filter plate. Cell were then washed with PBS, and stained with Live/Dead UV blue dead cell stain (Invitrogen). FcR block (BD Pharmingen) was then added, followed by B220 Cy7PE (BD Pharmingen), F4/80 Cy5PE (eBiosciences), CD80 PE-CF594 (BD Pharmingen), CD11c PE (BD Pharmingen), Ly-6C Cy7APC (BD Pharmingen), CD11b Ax700 (BioLegend), CD8 BV785 (BD Horizon), Ly-6G BV605 (BD Horizon), CD3e BV510 (BD Horizon), NK1.1 BV510 (BD Horizon), and CD19 BV421 (BD Horizon). Cells were then washed twice with PBS and resuspended in PBS + 0.5% PFA; events were acquired on an LSR Fortessa X-50 (BD Biosciences). Cytometry data were analyzed using FlowJo software (Tree Star), PESTLE (Mario Roederer, Vaccine Research Center, NIAID) and SPICE (6). Gating trees for all populations studied are described in S2 Fig.

**Confocal microscopy and histocytometry.** Popliteal LNs were harvested 4 hrs., 24 hrs., or 10 days after vaccination, embedded in O.C.T. (Sakura Fintek), and frozen at -80°C. 8 μm sections were cut by cryostat (American Histolabs) to get representative tissue cross-sections. Tissue sections were mounted on highly charged glass slides at stored at-80**°**C. Just before use, sections were thawed for 1hr at RT and then fixed with 1% PFA for 30 min. Tissue sections were then blocked with 10% rat serum in permeabilization buffer (0.3% Triton–X100, 1% BSA in PBS). Tissue sections were then stained with CD11b BV510 (clone M1/70, BioLegend); CD11c-BV421 (clone N418, BioLegend); IA/IE MHCII-Ax488 (clone M5-114.15.3, BioLegend); CD4-Ax546 (conjugated in-house); CD169 BV605 (clone 3D6.112, BioLegend); Ki67 BV421 (clone 11F6, BioLegend); GL7-Pacific Blue (clone GL7, BioLegend), with overnight incubation at 4**°**C in a humidified chamber. The following day, the slides were washed with PBS 3x, 20 min each at RT. Finally, jopro (Thermo-Fisher) was applied for 20 min. and slides were mounted with Fluoromount-G. Confocal images were acquired with Nikon confocal microscope (A1^+^) equipped with 40x 1.3 NA oil objective. Image acquisition was operated with NIS- Elements Advanced Research Software. Live spectral unmixing were performed using single color stained controls to correct for spectral spill over. Imaging analyses were done with Imaris v8.4.1. Multiplex histocytometry was performed using methods described before (7, 8). Briefly, imaging data sets were segmented using nuclear staining signal (jopro). Average voxel intensity for each fluorochrome was extrapolated after generation of iso-surface. Data (including position, sphericity and volume) were then exported as csv (comma separated value) file and analyzed in FlowJo v10.4.1.

*ELISA*

Antibody titers from serum were measured as previously reported (5). For mouse studies, V3 peptides were coated directly onto Immulon 4 HBX plates (Thermo Scientific). For NHP studies, biotinylated forms of the Man9V3 and aglycone V3 peptides were coated onto streptavidin coated high capacity plates (Pierce). For JRFL ELISAs, SOSIP trimers were captured on plates coated with lectin from *Galanthus nivalis* (Sigma-Aldrich). Reciprocal endpoint titers were determined as the titer at which the curve crossed the positive threshold of OD_450_=0.2. Because the lowest serum dilution was 1:20, the limit of detection is 20; samples that did not register as positive at the 1:20 dilution are automatically graphed as a titer of 10. For antibody mapping, mAbs were applied to ELISA plates in 10x dilutions starting at 5 μg/mL, diluted in blocking buffer.

*CH0848 binding and blocking assays*

**Binding ELISA***.* SOSIP binding ELISAs were conducted in 384 well ELISA plates (Costar) coated with 2 ug/ml anti-AVI_tag antibody (Avidity #I1514)* in 0.1 M sodium bicarbonate overnight at 4°C. Plates were washed (PBS, 0.1% Tween 20) and blocked with assay diluent (PBS containing 4% (w/v) whey protein, 15% normal goat serum, 0.5% Tween 20, 0.05% NaN_3_). AVI_tagged SOSIPs were added at 2 μg/mL in assay diluent for 60 min at RT, followed by washing and addition of serially diluted samples for 90 min at RT. Plates were washed and 10 μL HRP conjugated mouse anti-rhesus secondary antibody (Southern Biotech) diluted in assay diluent (without azide), was incubated for 60min at RT, washed and detected with 20 µl SureBlue Reserve (KPL) for 15min at RT. Reaction was stopped with the addition of 20 μl 0.33 N HCL. Plates were read at 450nm. *If a SOSIP lacked an AVI_tag, it was captured by coating 2 μg/ml human anti-SOSIP antibody, PGT151, otherwise following the same protocol.

**Blocking assay:** 384 well ELISA plates (Costar) were coated with Env overnight at 4°C and blocked with assay diluent (PBS containing 4% (w/v) whey protein, 15% normal goat serum, 0.5% Tween 20, 0.05% NaN_3_) for 60 min at RT. Samples were diluted 1:50 in assay diluent and incubated in triplicate wells for 90 min. 10 μl biotinylated target mAb was added at the EC50 (determined by a direct binding of biotinylated-mAb) for 60 min. at RT. Biotin-mAb binding was detected with streptavidin-HRP (Thermo Scientific) at 1:30,000 (60 min. at RT) followed by 20 μl SureBlue Reserve (KPL). Reaction was stopped with 0.33N HCL and plates were read at 450 nm. After background subtractions, percent blocking was calculated as follows: 100-((sera triplicate mean/no blocking control mean)*100).

*Mouse T cell responses*

Splenocytes were harvested and stimulated *ex vivo* as previously reported (9). Briefly, cells were incubated with anti-CD28, brefeldin A with either: 2 μg/ml V3 peptide, 2 μg/ml PADRE peptide, PMA/ionomycin (positive control), or media alone (negative control) at 37°C for 6 h, then overnight at 4°C. For intracellular cytokine staining, cells were washed, incubated with Aqua Blue live/dead stain (Invitrogen), then with FcR block (BD Pharmingen), followed by staining for surface markers (CD4 Ax700, BD Pharmingen and CD8 APC-Cy7, BioLegend) in PBS+serum. Cells were then permeabilized and stained intracellularly (CD3 Cy5PE, TNFα PE-Cy7, IL-2 PE, IFN-γ APC, all from BD Pharmingen), followed by washing and fixing in PBS + 0.5% PFA. Flow cytometry was performed as above. Gating trees for cytokine positive populations are depicted in S1B Fig.

*Virus stocks and neutralization assays*

HIV-1 Env pseudoviruses were prepared by transfecting 293T cells (6×10^6^ cells in 50 ml growth medium in a T-175 culture flask) with 10 μg of rev/env expression plasmid and 30 μg of an env-deficient HIV-1 backbone vector (pSG3ΔEnvelope), using Fugene 6 transfection reagent (Invitrogen). Pseudovirus-containing culture supernatants were harvested 2 days after transfection, filtered (0.45 μm), and stored at −80°C or in the vapor phase of liquid nitrogen. Neutralization was measured using HIV-1 Env pseudoviruses to infect TZM-bl cells as described previously (10, 11). Briefly, 40 μl of pseudovirus was incubated for 30 min at 37°C with 10 μl of serially diluted test antibody in duplicate wells of a 96-well flat-bottomed culture plate. To keep assay conditions constant, sham medium was used in place of antibody in control wells. The pseudovirus input was set at a multiplicity of infection of approximately 0.01, which generally results in 100,000 to 400,000 relative light units (RLU) in a luciferase assay (Bright Glo; Promega, Madison, WI). Values were interpolated from duplicate relative light unit measurements, and in some cases are means of duplicate runs. Neutralization curves were fit by nonlinear regression using a 5-parameter hill slope equation as previously described (12). The 50% inhibitory concentrations (IC_50_) were reported as the antibody concentrations required to inhibit viral entry by 50%.

*B cell immunophenotyping*

NHP PBMC were stained with Aqua Blue live/dead stain (Invitrogen), then with CD20 Ax700-PE (BD Pharmingen), IgM PECy5 (BD Pharmingen), CD3 Cy7APC (BD Pharmingen), IgG APC (BD Pharmingen), CD14 BV785 (Bio Legend), and CD8 V450 (BD Pharmingen). Additionally, vaccine-specific responses were identified using aglycone V3-biotin/SA-FITC and Man_9_V3-biotin/SA-PE; SOSIP-specific responses were identified with BG505 SOSIP-biotin/SA-APC and IgG-PE (BD Pharmingen). Cells were washed and flow cytometry data were acquired as above.

*Antibody cloning*

Antigen-specific cells were sorted using a BD FACSAria II running FACSDiva software. Cell were sorted at 1 cell/well into lysis buffer and RNA was reverse transcribed as previously reported (13). IgG heavy and light chains were amplified by PCR and subcloned into expression vectors as previously reported (13). For protein expression, Expi293F cells were transfected using an ExpiFectamine 293 kit according to the manufacturer’s instructions (Gibco). 7.5 ug each of heavy and light chain plasmids were transfected into 30 mL cells seeded at 2-3 x 10^6^ cells/mL in 50 conical tubes and incubated at 37°C, 10% CO_2_, 230 rpm shaking for 4-5 days. Antibodies were purified by passing clarified culture supernatants over a protein A agarose column (GE Healthcare), concentrating the eluate in Amicon Ultra-15 columns (Millipore), and buffer exchanging to PBS using Zeba spin columns (Pierce) according to the manufacturers’ instructions.

*Ig sequence analysis*

Heavy chain sequences from the cloned mAbs were analyzed using SONAR (14) employing the Ramesh *et al* (15) rhesus macaque immunoglobulin database as the reference library for V_H_, D_H_ and J_H_ gene assignments. The V_H_ divergence from germline was then calculated at the nucleotide level. HCDR3 lengths were calculated according to the IMGT definition (16).

**References:**

1. Ulbrich K*, et al.* (2000) Polymeric drugs based on conjugates of synthetic and natural macromolecules. I. Synthesis and physico-chemical characterisation. *Journal of controlled release : official journal of the Controlled Release Society* 64(1-3):63-79.

2. Šubr VK, L.; Strohalm, J.; Etrych, T.; and Ulbrich, K. (2013) Synthesis of Well-Defined Semitelechelic Poly[N-(2-hydroxypropyl)methacrylamide] Polymers with Functional Group at the α-End of the Polymer Chain by RAFT Polymerization. *Macromolecules* 46(6):2100–2108.

3. Shukla NM*, et al.* (2010) Syntheses of fluorescent imidazoquinoline conjugates as probes of Toll-like receptor 7. *Bioorg Med Chem Lett* 20(22):6384-6386.

4. Lynn GM*, et al.* (2015) In vivo characterization of the physicochemical properties of polymer-linked TLR agonists that enhance vaccine immunogenicity. *Nat Biotechnol* 33(11):1201-1210.

5. Francica JR*, et al.* (2015) Analysis of immunoglobulin transcripts and hypermutation following SHIV(AD8) infection and protein-plus-adjuvant immunization. *Nature communications* 6:6565.

6. Roederer M, Nozzi JL, & Nason MC (2011) SPICE: exploration and analysis of post-cytometric complex multivariate datasets. *Cytometry A* 79(2):167-174.

7. Petrovas C*, et al.* (2017) Follicular CD8 T cells accumulate in HIV infection and can kill infected cells in vitro via bispecific antibodies. *Sci Transl Med* 9(373).

8. Amodio D*, et al.* (2018) Quantitative Multiplexed Imaging Analysis Reveals a Strong Association between Immunogen-Specific B Cell Responses and Tonsillar Germinal Center Immune Dynamics in Children after Influenza Vaccination. *Journal of immunology* 200(2):538-550.

9. Quinn KM*, et al.* (2013) Comparative analysis of the magnitude, quality, phenotype, and protective capacity of simian immunodeficiency virus gag-specific CD8+ T cells following human-, simian-, and chimpanzee-derived recombinant adenoviral vector immunization. *Journal of immunology* 190(6):2720-2735.

10. Li M*, et al.* (2005) Human immunodeficiency virus type 1 env clones from acute and early subtype B infections for standardized assessments of vaccine-elicited neutralizing antibodies. *J Virol* 79(16):10108-10125.

11. Sarzotti-Kelsoe M*, et al.* (2014) Optimization and validation of the TZM-bl assay for standardized assessments of neutralizing antibodies against HIV-1. *Journal of immunological methods* 409:131-146.

12. Seaman MS*, et al.* (2010) Tiered categorization of a diverse panel of HIV-1 Env pseudoviruses for assessment of neutralizing antibodies. *J Virol* 84(3):1439-1452.

13. Mason RD*, et al.* (2016) Targeted Isolation of Antibodies Directed against Major Sites of SIV Env Vulnerability. *PLoS pathogens* 12(4):e1005537.

14. Schramm CA*, et al.* (2016) SONAR: A High-Throughput Pipeline for Inferring Antibody Ontogenies from Longitudinal Sequencing of B Cell Transcripts. *Frontiers in immunology* 7:372.

15. Ramesh A*, et al.* (2017) Structure and Diversity of the Rhesus Macaque Immunoglobulin Loci through Multiple De Novo Genome Assemblies. *Frontiers in immunology* 8:1407.

16. Lefranc MP*, et al.* (2003) IMGT unique numbering for immunoglobulin and T cell receptor variable domains and Ig superfamily V-like domains. *Dev Comp Immunol* 27(1):55-77.
